# Effectiveness of a Mouth Care Program Provided by Nursing Home Staff vs Standard Care on Reducing Pneumonia Incidence: A Cluster Randomized Trial

**DOI:** 10.1001/jamanetworkopen.2020.4321

**Published:** 2020-06-19

**Authors:** Sheryl Zimmerman, Philip D. Sloane, Kimberly Ward, Christopher J. Wretman, Sally C. Stearns, Patricia Poole, John S. Preisser

**Affiliations:** 1 The Cecil G. Sheps Center for Health Services Research, University of North Carolina at Chapel Hill; 2 School of Social Work, University of North Carolina at Chapel Hill; 3 Gillings School of Global Public Health, University of North Carolina at Chapel Hill; 4 Department of Family Medicine, School of Medicine, University of North Carolina at Chapel Hill

## Abstract

**Importance:**

Pneumonia affects more than 250 000 nursing home (NH) residents annually. A strategy to reduce pneumonia is to provide daily mouth care, especially to residents with dementia.

**Objective:**

To evaluate the effectiveness of Mouth Care Without a Battle, a program that increases staff knowledge and attitudes regarding oral hygiene, changes mouth care, and improves oral hygiene, in reducing the incidence of pneumonia among NH residents.

**Design, Setting, and Participants:**

This pragmatic cluster randomized trial observing 2152 NH residents for up to 2 years was conducted from September 2014 to May 2017. Data collectors were masked to study group. The study included 14 NHs from regions of North Carolina that evidenced proportionately high rehospitalization rates for pneumonia and long-term care residents. Nursing homes were pair matched and randomly assigned to intervention or control groups.

**Intervention:**

Mouth Care Without a Battle is a standardized program that teaches that mouth care is health care, provides instruction on individualized techniques and products for mouth care, and trains caregivers to provide care to residents who are resistant and in special situations. The control condition was standard mouth care.

**Main Outcomes and Measures:**

Pneumonia incidence (primary) and hospitalization and mortality (secondary), obtained from medical records.

**Results:**

Overall, the study enrolled 2152 residents (mean [SD] age, 79.4 [12.4] years; 1281 [66.2%] women; 1180 [62.2%] white residents). Participants included 1219 residents (56.6%) in 7 intervention NHs and 933 residents (43.4%) in 7 control NHs. During the 2-year study period, the incidence rate of pneumonia per 1000 resident-days was 0.67 and 0.72 in the intervention and control NHs, respectively. Neither the primary (unadjusted) nor secondary (covariate-adjusted) analyses found a significant reduction in pneumonia due to Mouth Care Without a Battle during 2 years (unadjusted incidence rate ratio, 0.90; upper bound of 1-sided 95% CI, 1.24; *P* = .27; adjusted incidence rate ratio, 0.92; upper bound of 1-sided 95% CI, 1.27; *P* = .30). In the second year, the rate of pneumonia was nonsignificantly higher in intervention NHs. Adjusted post hoc analyses limited to the first year found a significant reduction in pneumonia incidence in intervention NHs (IRR, 0.69; upper bound of 1-sided 95% CI, 0.94; *P* = .03).

**Conclusions and Relevance:**

This matched-pairs cluster randomized trial of a mouth care program compared with standard care was not effective in reducing pneumonia incidence at 2 years, although reduction was found during the first year. The lack of significant results in the second year may be associated with sustainability. Improving mouth care in US NHs may require the presence and support of dedicated oral care aides.

**Trial Registration:**

ClinicalTrials.gov Identifier: NCT03817450

## Key Points

**Question:**

Can nursing home staff reduce pneumonia by providing mouth care to residents?

**Findings:**

In this pragmatic cluster randomized trial including 2152 residents from 14 paired nursing homes during 2 years, there was no statistically significant difference in the primary outcome of pneumonia incidence during 2 years. Adjusted post hoc analyses indicated a statistically significant reduction in pneumonia incidence during the first year.

**Meaning:**

The results of this study suggest that it is possible for nursing home staff to reduce pneumonia, but doing so will require sustained efforts.

## Teaser

This cluster randomized trial evaluates the effectiveness of Mouth Care Without a Battle, a program that increases staff knowledge and attitudes regarding oral hygiene, changes oral care, and improves oral hygiene, in reducing the incidence of pneumonia among nursing home residents.

## Introduction

Those familiar with nursing home (NH) care will appreciate the perspective of a leader in geriatric dentistry: “I always say you can measure quality in a nursing home by looking in people’s mouths, because it’s one of the last things to be taken care of.”^1^ Related evidence indicates that 84% of dependent long-term care residents do not receive mouth care, and when nursing assistants do brush teeth, they spend an average of 16 seconds doing so, far below the 2-minute American Dental Association recommendation.^2,3^ Poor care translates to substantial oral debris, untreated decay, and gingivitis.^4,5,6^

Common barriers to mouth care in NHs are residents who resist care and staff who lack time and knowledge.^7^ In response, numerous initiatives have been developed and evaluated to improve care, including brushing by dental nurses,^8^ dental experts working with NH staff,^9^ and helping NH staff themselves provide better care.^10,11,12,13^ Common findings were that after training, staff were more knowledgeable and had better attitudes, residents were more likely to allow mouth care, and oral hygiene was improved.^8,9,10,11,12,13,14,15,16^ Therefore, it is feasible to improve the oral hygiene of NH residents. What is lacking is system-level change.

One driver for system-level change would be if mouth care reduced pneumonia. Pneumonia resulting from aspiration of microorganisms in the oral cavity was suggested 30 years ago,^17^ and numerous studies have demonstrated this association.^18,19^ The association is especially concerning for NH residents, who have substantially more oral colonization of respiratory pathogens than other adults^20,21^ and for whom pneumonia is the second most common infection, affecting at least 250 000 residents annually.^22^

A 2018 systematic review of mouth care and pneumonia^23^ identified 4 studies, all of professional vs usual care, and found low-level evidence that professional care reduced mortality from pneumonia, but results on pneumonia incidence were inconclusive.^23^ More pragmatic studies using NH staff have evidenced variable results, with null results being found in studies with small and nonrepresentative samples.^24,25,26^

A common risk factor for poor oral hygiene in NHs is dementia; these individuals often resist care, and the prevalence of residents with dementia in NHs is high (approximately 61% have moderate or severe cognitive impairment).^27,28,29,30^ Consequently, what is needed is a pragmatic intervention that includes a focus on care for individuals with dementia. This project evaluated such a program, Mouth Care Without a Battle (MCWB), which teaches NH staff techniques to provide mouth care, especially to residents who resist care. Previous studies demonstrated its effect on staff knowledge and care and resident oral hygiene.^12,13,14^ This article reports the results of a 2-year pragmatic cluster randomized trial conducted in 14 NHs, studying pneumonia incidence (primary outcome) and hospitalization and mortality (secondary outcomes). A cluster randomized trial design was chosen because the target of the intervention was the NH system and the desire was to evaluate a new standard of care and its effect on resident outcomes; also, it avoided potential contamination from spillover effects had randomization been at the level of the individual.

## Methods

### Design, Settings, and Randomization

We invited 16 NHs in areas of North Carolina that evidenced proportionately high rehospitalization rates for pneumonia and of long-term care residents; the administrator of 1 NH refused, and another was not enrolled because there was no NH of a similar size for matching (Figure). The remaining NHs were matched in pairs based on size and 6-month pneumonia incidence rate prior to study onset. Within each pair, 1 NH was randomized to MCWB by random number generation conducted by the project statistician. In these NHs, staff were trained and supported to provide daily mouth care for 2 years following MCWB practices. Medical records of all residents in and admitted to the NHs during the study period were abstracted, and those in MCWB NHs were expected to receive mouth care accordingly. A waiver of consent to abstract medical records was granted by the Office of Human Research Ethics of the University of North Carolina at Chapel Hill, which approved all study procedures; the Office of Human Research Ethics determined that the amount of data to be collected was limited and that it was impracticable to obtain consent for all residents. The study was registered on ClinicalTrials.gov after data collection was complete because of a change in the National Institutes of Health clinical trial rules that were in effect at the start of the study. The study protocol is available in Supplement 1.

**Figure.**
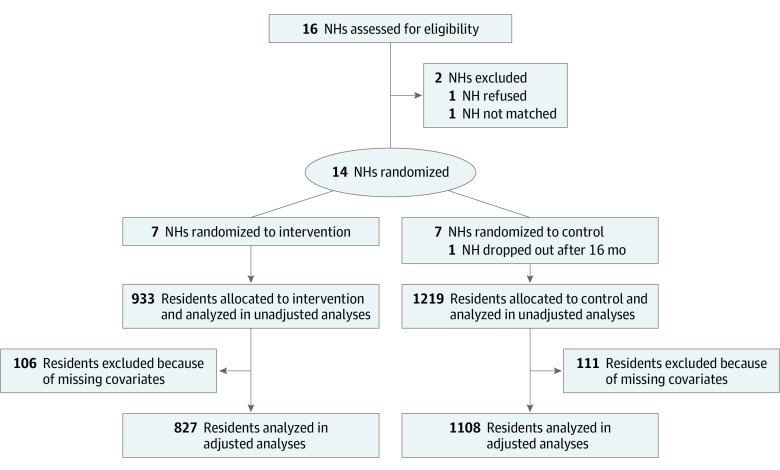
Flow Diagram of Nursing Home (NH) and Resident Enrollment and Analysis

### Intervention

Mouth Care Without a Battle was developed by an interdisciplinary team of clinician scientists and is described elsewhere.^12,13^ It highlights that mouth care is health care; provides information about techniques and products to clean and protect the teeth, tongue, gums, and dentures; informs care in special situations; and provides guidance when caring for people who are resistant to mouth care.^31^ For example, MCWB teaches the jiggle sweep technique to remove plaque from the teeth; the use of interdental brushes to clean between the teeth; the benefit of nonalcohol rinse instead of toothpaste for residents who have difficulty swallowing and of chlorhexidine for residents with significant gingivitis; how to steady a loose tooth when cleaning other teeth; the importance of not using toothpaste to clean dentures; and techniques, such as gentle massage of the cheek and jaw and singing, to encourage residents to open their mouths.

The intervention included 3 in-service trainings provided by a specialist in dementia care and dental hygiene at study onset and monthly support visits over 2 years; at 12 months, a second in-service training was conducted. All nursing assistants, nurses, and administrative staff were invited to the training. In each NH, a nursing assistant was identified as a dedicated oral care aide; they provided staff support, trained new staff, and cared for residents who required the most time. Their salary was provided by the research grant. The control condition was standard mouth care.

### Outcomes and Masking

The primary outcome was incidence of pneumonia per 1000 resident-days. All resident medical records were abstracted quarterly during the study period; pneumonia was considered present based on a diagnosis of pneumonia recorded in nurse or provider notes. The validity of this strategy was based on the review of 1119 medical records early in the project, which compared select signs and symptoms of the McGreer criteria,^32^ antibiotic prescribing, and recorded diagnosis of pneumonia. Based on 108 potential pneumonia cases, diagnosis detected 107 (99.1%); modified McGreer criteria, 84 (77.8%); and antibiotic prescription, 47 (43.5%).^33^

Secondary resident outcomes included incidence of all-cause hospitalization, hospitalization due to pneumonia, and mortality, also obtained from medical records. Outcomes related to staff self-efficacy and mouth care provision are reported elsewhere.^34,35^

Additional data included resident- and site-level baseline characteristics. Resident data were abstracted from the Minimum Data Set 3.0 form, including 35 variables empirically associated with oral health in other studies (eg, demographic characteristics, eating dependency, health problems, medications, and swallowing, feeding, and dental issues). Data on NHs (eg, organizational structure, oral hygiene practices, staffing) were obtained by interview with administrators. Data collection was conducted from September 2014 to May 2017 by research assistants masked to study group.

### Sample Size

The estimate of statistical power was based on pneumonia incidence over 2 years of follow-up. Using previous research, it assumed a pneumonia rate of 2 cases per 1000 resident-days.^36,37^ With an average of 102 residents per NH, it was estimated that 74 460 resident-days and 149 pneumonia cases would be observed per control site, resulting in 80% power to detect a pneumonia incidence reduction of 19% with a prespecified, 1-sided test for α = .05. Power calculations assumed an overdisperson factor for NH level rates of 3.0 to account for clustering; they were further based on an unmatched analysis, assuming they would be conservative and hence would provide justification for the planned matched analyses.

### Statistical Analysis

Resident-level incidence counts were summed and rates calculated by dividing event counts by days of exposure (ie, days in the NH during 2 years). From these rates, aggregate site-level rates were calculated and aggregated into group-level rates.

Analysis of intervention effectiveness computed incidence rate ratios (IRRs) to compare groups, with an IRR less than 1.00 indicating a reduction in pneumonia among MCWB NHs compared with control NHs. The study prespecified 1-sided tests for intervention effectiveness with respect to the primary outcome due to assumptions that MCWB would not increase pneumonia. Statistical significance was set at *P* < .05. Analyses were conducted in Stata version 15.0 (StataCorp) and SAS statistical software version 9.4 (SAS Institute).

The prespecified primary test of pneumonia rates was calculated using unadjusted IRRs after 2 years of follow-up and determining *P* values for the difference between groups based on the permutation distribution of the paired *t* statistic for the matched-pair differences in log rates.^38^ Under this framework, the null permutation distribution results from the 128 possible sign inversions of the 7 differences in log rates. It accounts for the fact that NHs were randomized within pairs and that there were 128 equally likely ways that intervention assignments could have occurred. The rank of the paired *t* statistic among all 128 possible statistics provided statistical significance. Separate post hoc tests were calculated for the first and second years of follow-up.

Secondarily, covariate-adjusted IRRs were derived and compared using permutation tests to account for baseline imbalances in resident-level characteristics between groups.^39,40^ First, the prognostic value of the 35 Minimum Data Set variables were studied individually via negative binomial models with NH-specific fixed-effect intercepts; resident exposure necessitated an offset variable. Then, 15 covariates that had *P* ≤ .15 were modeled jointly using another negative binomial model but with NH-pair specific fixed-effect intercepts. A final set of covariates was derived using a backward selection process, with the least significant covariates being removed iteratively until all remaining covariates (n = 7) were significant (ie, *P* < .05). A total of 435 residents (20.2%) had missing values on some covariates used in adjusted analyses, and values were imputed (median or mode) for residents with 3 or fewer missing values; if more than 3 values were missing, the case was excluded from analysis. Nursing home–level residuals between log observed and log estimated pneumonia rates were determined by dividing the sum of fitted log pneumonia counts over residents in each NH by their total bed-days. Covariate-adjusted permutation tests were applied to the paired differences between residuals from intervention and control NHs, and covariate-adjusted IRRs were calculated as the geometric mean of the 7 NH pair-specific rate ratios.

Third, a complementary model-based approach to testing intervention effectiveness was conducted using mixed-effects negative binomial models with a dichotomous fixed effect for group, fixed effects for the same covariates as described previously, a random effect for NH site, and an offset for log days of exposure. While this approach has stronger statistical assumptions compared with the permutation test and ignores the matched pair design, it effectively weights the contribution of NHs relative to their numbers of observations. This model-based approach, including the same covariates, was used to test intervention effectiveness for the secondary outcomes with prespecified 2-sided tests, with *P* < .05 as the level of statistical significance.

## Results

### Nursing Home Characteristics

Participants included 1219 residents (56.6%) in the 7 intervention NHs and 933 residents (43.4%) in the 7 control NHs. The Figure displays the flow of study enrollment and analysis; 1 intervention site participated for only 16 months, so data for its matched home were analyzed only up to 16 months. Table 1 presents the characteristics of the 14 NHs. Most (11 [78.6%]) were for-profit and had a mean (SD) of 105.0 (24.9) beds. No NH characteristics differed largely between groups, although intervention homes had a larger mean (SD) percentage of residents who required attention for behavior (21.4% [19.1%] vs 7.1% [4.9%]).

**Table 1.** Baseline Nursing Home and Resident Characteristics by Group

Characteristic	No. (%)		
	Total	Control	Intervention
Nursing home characteristic	14	7 (50.0)	7 (50.0)
For profit	11 (78.6)	5 (71.4)	6 (85.7)
Time in operation, mean (SD), y	27.2 (10.5)	25.3 (12.4)	29.1 (8.8)
Licensed beds, mean (SD), No.	105.0 (24.9)	104.3 (25.1)	105.7 (26.7)
Private rooms, mean (SD), %	30.0 (28.3)	25.1 (20.2)	35.0 (35.6)
Resident case-mix, mean (SD), %			
Dementia diagnosis	60.0 (13.0)	56.6 (14.3)	63.4 (11.6)
Require staff attention for behavior	14.3 (15.3)	7.1 (4.9)	21.4 (19.1)
Medicaid case-mix	61.6 (20.2)	62.1 (15.0)	61.0 (25.7)
Medicare case-mix	22.1 (14.2)	20.4 (16.8)	23.7 (12.1)
Nursing home star rating, mean (SD)^a^	3.7 (1.4)	3.6 (1.5)	3.9 (1.4)
Staffing, mean (SD)			
RN and LPN minutes per resident day	108.7 (40.4)	112.6 (48.5)	104.9 (34.0)
CNA minutes per resident day	152.2 (48.3)	145.6 (27.4)	158.9 (64.8)
Oral health services			
On-site dentist visits	7 (50.0)	2 (28.6)	5 (71.4)
On-site hygienist visits	2 (14.3)	0	2 (28.6)
Previous 6-month pneumonia rate, mean (SD)^b^	0.77 (0.29)	0.73 (0.31)	0.82 (0.27)
Resident characteristics^c^	2152	933 (43.4)	1219 (56.6)
Age at baseline, mean (SD), y	79.4 (12.4)	80.8 (11.8)	78.5 (12.8)
Women	1281 (66.2)	577 (69.9)	704 (63.5)
Race			
White	1180 (62.2)	510 (63.0)	670 (61.6)
African American	580 (30.6)	272 (33.6)	308 (28.3)
Other	138 (7.3)	28 (3.5)	110 (10.1)
Resident health conditions and care			
Chronic renal disease	240 (12.5)	71 (8.6)	169 (15.4)
Alzheimer disease or a related dementia	867 (46.3)	342 (42.1)	525 (49.6)
Malnutrition	40 (2.1)	14 (1.7)	26 (2.4)
Asthma or COPD	383 (19.9)	151 (18.4)	232 (21.1)
Recent weight loss	115 (6.2)	58 (7.2)	57 (5.3)
Swallowing issues	76 (4.2)	23 (2.8)	53 (5.4)
Feeding tube	104 (5.4)	44 (5.4)	60 (5.5)
Hospice care	104 (5.5)	58 (7.1)	46 (4.2)
Dental issues			
No teeth or fragments	197 (10.3)	74 (9.0)	123 (11.2)
Likely cavities	75 (3.9)	30 (3.7)	45 (4.1)
Other dental issues	24 (1.4)	15 (1.9)	9 (0.9)
Medication use and vaccinations			
Antibiotic use at baseline	343 (17.9)	145 (17.6)	198 (18.1)
Flu vaccine documented this year	777 (44.6)	319 (41.4)	458 (47.1)
Pneumococcal vaccine up to date	997 (52.8)	449 (54.6)	548 (51.3)
Eating dependency			
Independent eating	428 (22.4)	175 (21.3)	253 (23.3)
Supervised eating	788 (41.3)	343 (41.8)	445 (40.9)
Limited assistance	154 (8.1)	87 (10.6)	67 (6.2)
Extensive assistance	373 (19.5)	131 (16.0)	242 (22.2)
Total dependence	166 (8.7)	85 (10.4)	81 (7.4)

Abbreviations: CNA, certified nursing assistant; COPD, chronic obstructive pulmonary disease; LPN, licensed practical nurse; RN, registered nurse.

^a^ Nursing home star rating is the Centers for Medicare & Medicaid Services overall 5-star rating, which ranges from 1 (low) to 5 (high).

^b^ Rate per 1000 resident-days.

^c^ Due to missing data, resident sample ranges from 1742 to 1934.

### Resident Characteristics

Table 1 presents the baseline characteristics of the 2152 residents. They had a mean (SD) age of 79.4 (12.4) years of age and included 1281 (66.2%) women and 1180 (62.2%) white residents. Despite randomization, there were several differences between groups. Of potential importance to mouth care and pneumonia risk, residents in intervention homes were more likely to have chronic renal disease (169 [15.4%] vs 71 [8.6%]), Alzheimer disease or a related dementia (525 [49.6%] vs 342 [42.1%]), and documentation of receipt of the flu vaccine (458 [47.1%] vs 319 [41.4%]). The mean (SD) length of stay in the study was 342.1 (264.8) days.

### Pneumonia Incidence

During the matching period, pneumonia incidence rates ranged from 0.35 to 1.26 per 1000 resident-days, with a postrandomization mean (SD) in control and intervention NHs of 0.73 (0.31) and 0.82 (0.27), respectively. Table 2 presents pneumonia incidence rates and results of the primary and secondary analytic approaches using unadjusted and covariate-adjusted IRRs. The study observed 509 cases of pneumonia among 395 residents (182 [46.1%] in control; 213 [53.9%] in intervention). The 2-year rate was 0.72 per 1000 resident-days in the control group and 0.67 per 1000 resident-days in the intervention group. Neither the primary (unadjusted) nor secondary (covariate-adjusted) analyses found significant reduction in pneumonia due to MCWB in the full study period (unadjusted incidence rate ratio, 0.90; upper bound of 1-sided 95% CI, 1.24; *P* = .27; adjusted incidence rate ratio, 0.92; upper bound of 1-sided 95% CI, 1.27; *P* = .30). In year 2, the rate of pneumonia was higher in intervention homes than in control homes (0.65 per 1000 resident-days vs 0.51 per 1000 resident-days), although this difference was not statistically significant; post hoc unadjusted and adjusted 2-sided 95% CIs for the intervention effect IRRs, both estimated as 1.19 (Table 2) were 0.66 to 2.15 and 0.62 to 2.25, respectively.

**Table 2.** Pneumonia Incidence Rates, Ratios, and Permutation Tests for Intervention Effectiveness

Follow-up	Observed incidence rate per 1000 resident-days^a^		Unadjusted IRR^b^		Covariate-adjusted IRR^b^^,^^c^	
	Control NHs	Intervention NHs	IRR (1-sided 95% CI)	*P* value	IRR (95% CI)	One-sided *P* value
Years 1 and 2	0.72	0.67	0.90 (1.24)	.27	0.92 (1.27)	.30
Year 1 only	0.91	0.68	0.73 (1.08)	.09	0.74 (0.99)	.04
Year 2 only	0.51	0.65	1.19 (1.90)	.78	1.19 (1.98)	.75

Abbreviations: IRR, incidence rate ratio; NH, nursing home.

^a^ Observed rates (1000 resident-days) ignoring NH identities.

^b^ Geometric mean of the 7 NH pair-specific rate ratios (intervention rate/control rate), 1-sided asymptotic 95% confidence intervals, and *P* values from a priori 1-sided α = .05 tests based on the permutation distribution of the paired *t* statistic for the paired differences in log rates.

^c^ Adjusted IRRs adjust for 7 resident-level covariates, as follows: age at baseline, no eating support, asthma or chronic obstructive pulmonary disease, feeding tube, antibiotic medication, flu vaccine documented this year, and pneumococcal vaccine up to date.

In the unadjusted year 1 analysis, the effect of the intervention on pneumonia incidence was not statistically significant (IRR, 0.73; upper bound of 1-sided 95% CI, 1.08; *P* = .09). In the covariate-adjusted analysis at year 1, the intervention significantly reduced pneumonia incidence (IRR, 0.74; upper bound of 1-sided 95% CI, 0.99; *P* = .04).

Table 3 presents the tertiary approach using model-adjusted pneumonia IRRs. As above, neither the full study period nor the post hoc year 2–only models resulted in a statistically significant intervention effect. However, post hoc results for year 1 indicated a significant 31% reduction in pneumonias among NHs that participated in MCWB compared with controls (IRR, 0.69; upper bound of 1-sided 95% CI, 0.94; *P* = .03).

**Table 3.** Model-Adjusted Pneumonia IRRs for Intervention Effectiveness^a^

Variable	Years 1 and 2 (n = 1921)		Year 1 (n = 1606)		Year 2 (n = 1284)	
	IRR (95% CI)	*P* value	IRR (95% CI)	*P* value	IRR (95% CI)	*P* value
Intervention group^b^	0.84 (1.12)	.16	0.69 (0.94)	.03	1.27 (1.75)	.90
Age	1.02 (1.01-1.03)	<.001	1.02 (1.01-1.03)	.001	1.01 (1.00-1.03)	.11
No eating support	0.31 (0.11-0.85)	.02	0.21 (0.05-0.92)	.04	0.52 (0.15-1.85)	.31
Asthma or COPD	2.11 (1.63-2.73)	<.001	2.06 (1.53-2.77)	<.001	1.86 (1.27-2.71)	.001
Feeding tube	2.91 (1.90-4.46)	<.001	2.67 (1.67-4.26)	<.001	2.25 (1.23-4.13)	.009
Antibiotic medication	1.57 (1.19-2.06)	.001	1.66 (1.21-2.26)	.001	1.39 (0.93-2.07)	.11
Flu vaccine	0.74 (0.59-0.93)	.01	0.78 (0.59-1.03)	.08	0.67 (0.48-0.94)	.02
Pneumococcal vaccine	1.25 (0.99-1.59)	<.001	1.39 (1.05-1.84)	<.001	1.01 (0.72-1.42)	.94
Dispersion parameter	1.21	NA	0.75	NA	1.33	NA
Random-effects variance	0.05	NA	0.6	NA	0.03	NA

Abbreviations: COPD, chronic obstructive pulmonary disease; IRR, incidence rate ratio; NA, not applicable.

^a^ Results based on resident-level negative binomial regressions with random effects to adjust for clustering within nursing homes and offsets to account for the number of resident days of exposure, with asymptotic 95% CIs.

^b^ One-sided test *P* values and the upper bound of 95% CIs for the intervention group effect.

### Any-Cause Hospitalization, Hospitalization Due to Pneumonia, and Mortality Incidence

Table 4 presents the model-adjusted approach to IRRs applied to data from year 1 only, given that there was no significant difference in pneumonia incidence after the first year. The year 1 observed rates (event counts) were 2.08 (351) per 1000 resident-days in the control group and 1.89 (410) per 1000 resident-days in the intervention group for any-cause hospitalization; 0.28 (48) per 1000 resident-days in the control group and 0.24 (53) per 1000 resident-days in the intervention group for hospitalization due to pneumonia; and 0.71 (120) per 1000 resident-days in the control group and 0.56 (122) in the intervention group for mortality incidence. While all 3 IRR estimates were less than 1.0, the results were not statistically significant.

**Table 4.** Model-Adjusted Year 1 IRRs for Effects of Intervention and Covariates on Any-Cause Hospitalization, Hospitalization Due to Pneumonia, and Mortality

Variable	Any-cause hospitalization (n = 1606)^a^		Hospitalization due to pneumonia (N = 1606)^b^		Mortality (n = 1606)^a^	
	IRR (95% CI)	*P* value	IRR (95% CI)	*P* value	IRR (95% CI)	*P* value
Intervention group	0.94 (0.66-1.34)	.73	0.84 (0.57-1.26)	.41	0.83 (0.61-1.12)	.23
Age	0.98 (0.98-0.99)	.001	1.02 (1.00-1.04)	.03	1.05 (1.03-1.06)	<.001
No eating support	0.64 (0.29-1.42)	.27	0.38 (0.05-2.77)	.34	0.59 (0.20-1.76)	.34
Asthma or COPD	1.68 (1.31-2.15)	<.001	2.10 (1.34-3.27)	.001	2.19 (1.53-3.13)	<.001
Feeding tube	1.96 (1.32-2.89)	.001	4.11 (2.36-7.14)	<.001	2.33 (1.26-4.30)	.007
Antibiotic medication	1.79 (1.39-2.31)	<.001	1.64 (1.03-2.62)	.04	1.14 (0.78-1.69)	.50
Flu vaccine	0.66 (0.53-0.81)	<.001	0.71 (0.47-1.06)	.10	0.77 (0.57-1.05)	.10
Pneumococcal vaccine	0.96 (0.77-1.20)	.72	1.01 (0.67-1.52)	.96	0.80 (0.58-1.10)	.16
Dispersion parameter	1.47	NA	NA	NA	1.12	NA
Random effects variance	0.08	NA	<0.0001	NA	0.004	NA

Abbreviations: COPD, chronic obstructive pulmonary disease; IRR, incidence rate ratio; NA, not applicable.

^a^ Negative binomial resident-level regressions with random effects to adjust for clustering within nursing homes and offsets to account for the number of resident days of exposure, with asymptotic 2-sided 95% CIs.

^b^ Poisson resident-level regressions with random effects to adjust for clustering within nursing homes and offsets to account for the number of resident days of exposure, with asymptotic 2-sided 95% CIs.

## Discussion

To our knowledge, this study is the first to examine whether a pragmatic program of mouth care provided by NH staff (ie, MCWB) reduces pneumonia. Neither the primary nor secondary analyses found a statistically significant reduction in pneumonia due to MCWB during 2 years, but covariate-adjusted post hoc analyses limited to the first year found a significant 26% to 31% reduction in pneumonia incidence. The study prespecified 1-sided tests for intervention effectiveness based on assumptions that MCWB would not increase pneumonia, which is appropriate when the hypothesis is of a dividing (ie, 1-sided) nature, as is the case when assessing effectiveness outcomes of MCWB.^41^ However, given the (nonsignificant) higher rate of pneumonia in the second year in intervention NHs, it is possible that altering oral flora can affect residents’ health in unpredictable ways.

We hypothesized a decrease in effectiveness during the study period due to challenges in sustainability, given indications that improvements in oral hygiene observed during the first year were not maintained in the second year, despite booster training and ongoing support; specifically, change from baseline peaked at 12 months and decreased at 20 and 24 months (eAppendix, eTables 1-5, and eFigure in Supplement 2).^35^ The issue of the sustainability of interventions in long-term care has been noted by others^42^ and requires attention.

All direct care staff were trained in MCWB and expected to provide mouth care; in addition, each NH had a dedicated oral care aide who supported other staff and cared for residents who required more time and attention (nearly always those with dementia). Another mouth care intervention that used dedicated aides found decreased mortality from pneumonia^24^ and similarly advocated for their use. We surmise that having a dedicated aide to care for persons with dementia who are at the greatest risk for poor oral hygiene^27,28,29,30^ is a critical component of success, in part because their care requires extra time.^12^

Another component that may have been key in reducing pneumonia in the first year is the MCWB recommendation to use chlorhexidine gluconate 0.12% for gingivitis, for which there is high-quality evidence of a large reduction in dental plaque when used in combination with tooth brushing.^43^ Our data indicate that chlorhexidine was used on 25% and 10% of resident-days in years 1 and 2, respectively (eTable 2 in Supplement 2), but we note that evidence regarding the benefits of chlorhexidine is controversial.^44,45,46^

### Limitations

This study has limitations. One limitation is generalizability, in that to be eligible, NHs had to evidence proportionately high rehospitalization rates for pneumonia and of long-term care residents; however, the NHs are similar to national averages in terms of for-profit ownership (79% in the study sample vs 69% in the US), mean bed size (105-106 beds in both), and overall mean Centers for Medicare & Medicaid Services star rating (3.7 vs 3.2). Also, pneumonia was ascertained based on medical record diagnosis, which could undercount or overcount cases (eg, due to incomplete documentation or misinterpretation of infiltrate on chest radiograph). However, it is highly pragmatic for use and has been found to be reliable and inclusive.^32^ Most importantly, it was the basis on which NHs were matched and monitored over time, and there is no reason to expect later bias between the study groups. In addition, we are cognizant of a recent critique that called for examination of the etiology of suspected pneumonia^47^ and agree that there is more to be learned.

## Conclusions

This matched-pairs cluster randomized trial of a mouth care program compared with standard mouth care was not effective in reducing pneumonia incidence at 2 years, although significant reduction was seen during the first year of the trial. The lack of significant results in the second year may be associated with sustainability. Improving mouth care in US NHs may require the presence and support of dedicated oral care aides.

## Notes

Supplement 1. **Trial Protocol**

Supplement 2. **eAppendix. **Supplementary Methods and Results **eTable 1. **Baseline Nursing Home Characteristics and Characteristics of Residents Participating in Oral Hygiene Assessments **eTable 2.** Mouth Care Provision Over Two Years Based on Mouth Care Logs for Residents in Mouth Care Without a Battle Nursing Homes **eTable 3.** Oral Hygiene of Repeated Cross-sectional Samples of Residents in 14 Nursing Homes During 2 Years **eTable 4.** Hierarchical Linear Mixed Model Estimates of Effects on Plaque and Gingival Oral Hygiene **eTable 5.** Two-Level Linear Mixed Model Estimates of Effects on Denture Plaque **eFigure.** Plaque, Gingival, and Denture Plaque Index Scores, Baseline Through 24 Months

Supplement 3. **Data Sharing Statement**
