# Supplementary material for: Effectiveness of a Mouth Care Program Provided by Nursing Home Staff vs Standard Care on Reducing Pneumonia Incidence: A Cluster Randomized Trial
Source: JAMA Netw Open. 2020 Jun 19;3(6):e204321. doi: 10.1001/jamanetworkopen.2020.4321 (PMC7305523; doi:10.1001/jamanetworkopen.2020.4321)
Supplement: Supplement 1. — Trial Protocol [file jamanetwopen-3-e204321-s001.pdf]

PROTOCOL

Overview. This two year matched pairs cluster randomized trial will be conducted in seven pairs of NHs chosen from 16 preselected homes that have high rates of hospitalization for pneumonia and resident characteristics related to pneumonia incidence. The seven pairs will be matched on pneumonia rate and size. One half of each pair will be randomly assigned to intervention (MCWB) or control, the latter receiving information related to risk factors for and incidence of hospitalization for falls. See Figure 1 for an overview of the project.

Figure 1. Project Overview

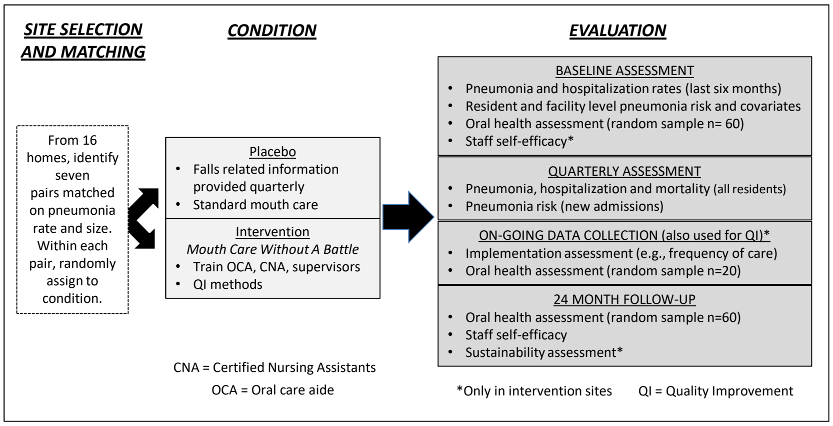

The intervention, MCWB, is a system-level approach to provide mouth care to all residents. It provides training to all CNAs and nursing supervisors, as well as the designation and specialized training of a CNA to serve as a dedicated, full-time Oral Care Aide (OCA) to provide mouth care to the residents who are at greatest risk for pneumonia and require specialized support to achieve good oral hygiene. QI techniques will be employed in intervention sites, involving ongoing review of mouth care and residents' oral hygiene.

Evaluation will begin with baseline assessment in all NHs, including pneumonia and hospitalization rates for the preceding six months, collection of resident- and facility-level pneumonia risks and covariates, and an oral health assessment for a random sample of 60 residents per NH. In intervention sites only (so as not to create a Hawthorne effect), baseline assessment also will include a measure of staff self-efficacy to provide mouth care. We will conduct quarterly chart reviews of pneumonia, hospitalization, and mortality rates for all residents across all 14 homes, as well as collect information related to the pneumonia risk of newly admitted residents. In intervention sites only, ongoing data will be collected related to program implementation and resident oral health status for purposes of QI as well as analysis. At the 24-month follow-up, a second oral health assessment will be conducted for a random sample of 60 residents per NH (intervention and control). Staff self-efficacy will be assessed in both control and intervention sites, and sustainability will be assessed.

The primary outcome is a matched pairs analysis to examine a facility-level difference in pneumonia incidence between intervention and control NHs. Secondarily, we will examine pneumonia incidence as it relates to resident-specific pneumonia risk and oral hygiene. Finally, to insure real world feasibility, the intervention's implementation, sustainability, costs, and cost-effectiveness, will be evaluated.

Site selection and matching. Because NH pneumonia rates are not publically available, a two-stage sampling process was used to derive the sample. First, we identified the six counties in North Carolina with the highest hospital readmission rates for pneumonia and other potentially avoidable conditions[66] that were also closest to the University of North Carolina. Within each county, we then used publically available data to identify 16 NHs that admit residents to those hospitals; have high re-admission rates (related to pneumonia incidence); have a case mix ≥ 50% Medicaid (a proxy for long-stay residents, desirable to limit attrition) and ≥ 20% Medicare case mix and/or rehospitalization rate;[67-68] and for which there was at least one other NH with a similar proprietary status (a characteristic related to quality)[69-70] and bedsize variance ≤ 25% (to assure a match for randomization). Table 1 (next page) provides select information about the resulting 16 NHs. While only 14 NHs will be enrolled, having a larger sampling pool than required allows us to create the best matched pairs. Letters of intent to participate, including agreement to randomization, are provided later in the proposal. Of note, the NH administrators were not provided details of each intervention; instead, they understand more generally that they will be changing care practices related to mouth care or falls prevention to reduce hospitalizations.

Matching will be done based on pneumonia rate (for the last six months) and size. Our goal will be to create seven pairs of NHs that differ in baseline pneumonia rate and bed size by less than 25% for both characteristics. Precedence will be given to matching on baseline pneumonia rate, so the matching criterion for bed size will be relaxed if necessary. Matches will be formed prior to randomization. Once matched, homes will be randomly assigned to condition and within pairs will start and end their participation at the same time.

Table 1. Characteristics of Nursing Homes Eligible for Participation (Select)

	Bedsize	Medicaid	Medicare	RN minutes[a]	LPN minutes[a]
Avante	110	71%	22%	40	64
Brian Center	99	67%	23%	31	58
Brookshire	80	55%	20%	40	73
Capital	125	72%	10%	30	56
Chapel Hill Rehab	120	64%	21%	47	41
Golden Living	122	71%	18%	46	44
Highland Acres	90	87%	8%	38	55
Laurels	120	70%	22%	68	61
Litchford Falls	90	57%	9%	39	33
Pembroke	84	73%	22%	31	47
Richmond Pines	105	83%	10%	24	52
Rockingham Manor	118	79%	16%	26	40
South Village	100	67%	17%	35	60
Universal	132	68%	9%	26	39
Wellington	80	58%	17%	23	64
Wilson Pines	86	58%	26%	25	65

[a] Minutes/resident/day.

Evaluating pneumonia risk. Each resident's pneumonia risk will be determined at study onset and as new residents are admitted. This information will be used in analyses and also made available to intervention NHs to determine which residents are in greatest need of care by the dedicated OCA (see section on OCA duties). While no empirical tool exists to estimate risk, a number of studies have identified risk factors for pneumonia in NH populations. Factors with adjusted risk odds ratios >2 for NH-acquired pneumonia include male gender, older age, increased physical impairment (or bedbound status), chronic obstructive pulmonary disease (COPD), sedative medication use, a history of aspiration, swallowing difficulty, and poor oral hygiene; absence of teeth (if not recently removed) has a protective effect.[27,71-74] All of these factors, with the exception of the quality of oral hygiene, are directly available from the Minimum Data Set (MDS), a resident-level assessment instrument mandated for use in virtually all NHs.[75] While the MDS does not assess the quality of oral hygiene, it includes items related to risk factors for poor oral health. Those with adjusted risk odds ratios >2 include older age, dementia (especially with concomitant agitation and/or resistive behavior), chronic kidney disease, and perceived need by staff for oral care.[42,76-78] From these studies we created the Pneumonia Risk Estimation Scorecard (see Figure 2). Of note, information obtained from the scorecard will be tested for predictive validity during the trial.

Figure 2. Pneumonia Risk Estimation Scorecard

Characteristic	MDS 3.0 Item(s)	Points
Gender: Male	A0800	1
Age: ≥ 85	A0900	1
Behavior interferes with care	E0500B or E0800	1
Extensive/total assistance in eating	G0110H	1
Alzheimer's disease/dementia	I14200 or I14800	1
Chronic renal disease	I1500	1
Chronic lung disease	16200	1
Coughing or choking during meals	K0100C	1
Dental problems on assessment	L0200 (not B or Z)	1
No teeth	L0200B	-1
Sedative or antipsychotic medication	N0400 (A,B or D)	1
	**Total score (-1 to 10)**	________

Control condition. The seven control NHs will continue to provide standard mouth care. In addition, the principal investigator and/or program coordinator will meet with their staff quarterly to provide information related to their residents' falls (incidence, severity), and the relationship of falls to resident mobility and hospitalization, as obtained from MDS information (Sections G, J) and chart reviews. Because falls are a condition of concern for NH administrators, we expect that our focus on falls will reduce the likelihood that staff at control homes will alter their behavior with regard to mouth care provision. At the end of the project period, CNAs, supervisors, and administrators in control NHs will receive training in MCWB.

Intervention condition. The intervention consists of MCWB in addition to established QI techniques we have used previously with success. All components of MCWB and QI will be discussed in a meeting between the research team (principal investigator Dr. Zimmerman, dementia specialist Dr. Sloane, dental expert Dr. Chen, and the project coordinator) and the NH staff (administrator, nursing staff, and selected others) before intervention onset, to discuss the unique circumstances necessary to maximize implementation and sustainability at that NH.

MCWB is a system-level, evidence-based, tested, approach to person-centered daily mouth care (tooth brushing, flossing, care of the gums, and denture care). It is appropriate for all individuals, but especially for persons with physical and cognitive impairment and who are at risk of pneumonia. MCWB combines best practices in oral hygiene care and dementia care to remove bacteria from the mouth even among individuals who resist care. MCWB includes CNA, supervisor, and administrator training, including the identification and training of a dedicated OCA selected from among the existing CNA staff. Below, we describe MCWB training, followed by a description of OCA selection and duties in greater detail.

The four modules of Mouth Care Without a Battle. MCWB training begins with viewing the program's four modules. Each module is presented on a digital video disk (DVD) (see Figure 3), three of which provide continuing education credits (administered by the North Carolina Nurses Association, Approval 8520). Content in each module includes:

- *Mouth care*: techniques and products to care for teeth, dentures, and gums, including the "jiggle and sweep" technique, interdental cleaners, and use of non-alcohol rinse instead of toothpaste (due to aspiration risk of toothpaste foam);
- *Behavioral challenges*: techniques to encourage people who are resistive (e.g., hit, bite, yell, spit), such as focusing on the person (not the task), establishing a daily routine, and personalizing strategies that work (e.g., sing a song to encourage the mouth to open, provide hand-over-hand guidance, gently massage the cheek and jaw);
- *Assessment and supervision* (oriented toward nurse supervisors): use of the modified Oral Health Assessment Tool (OHAT)[79] to identify conditions indicating the need for dental referral and to monitor care quality (i.e., it includes observation of the teeth, gums, and other areas); support provision for CNAs; care planning; and prescription products. Supervisors are taught to assess each resident's oral health status using the OHAT before the program of mouth care is initiated, and to consider making a referral for a dental evaluation if a resident exhibits problems in areas including the lips, gums/palate/inside of cheeks, teeth, dentures, tongue, or who have pain or dry mouth; and
- *Advocacy* (oriented to supervisors, administrators, regulators, and others): regulatory issues; organizational practices and policies to promote quality mouth care; monitoring mouth care quality.

Figure 3. MCWB DVD

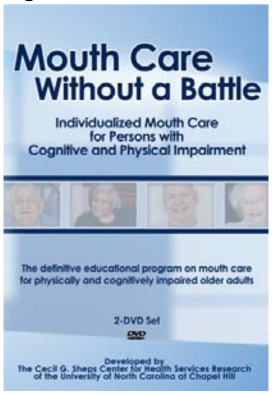

Mouth care products. Mouth care products are individualized for each resident, and do/may include a soft toothbrush; 0.12% chlorhexidine gluconate non-alcohol rinse (used for six weeks), followed by Biotene® alcohol-free moisturizing polymer oral rinse or -- for persons not at risk of aspiration -- toothpaste; gauze pads (to clean the gums); an interdental brush (to clean between the teeth); and 1.1% sodium fluoride paste. The use of these products is based on the best available evidence. Additional products include aerated caps to cover the brushes, plastic folders to hold each resident's supplies, disinfecting materials, and others. Information regarding mouth care products and their use is provided in Appendix 1. While recommended but not required, both the chlorhexadine rinse and the 1.1% sodium fluoride paste are prescription products, requiring that the medical director provide standing orders for their use. Such permission was granted by medical directors in our prior work with no hesitation. Further, while the CNAs and OCA are not certified medication technicians, the North Carolina Board of Nursing (NC BON) recognizes that these products do not carry systemic effects and can be safely used by CNAs trained in MCWB; in fact, the NC BON has provided a state-wide waiver of medication aide training for CNAs trained in MCWB procedures (see letter in Appendix 2). Also included in Appendix 2 is a letter of support from the North Carolina Health Facilities Association, which has a membership of 400 NHs. Their support will assure dissemination at the conclusion of a successful trial.

On-site training. In addition to viewing the DVD modules, MCWB includes eight weeks of on-site (but not full-time) training by an LPN / dental hygienist (Ms. Poole), supported by the project dementia specialist (Dr. Sloane) and principal investigator (related to organizational practices); all three were involved in the development and related training of MCWB. (It should be made clear that Ms. Poole will *not* conduct the oral health assessments detailed later). The CNAs, OCA, nurses, and administrator will be trained together in the first two modules, after which time training will be conducted either individually or in other groups specific to the related content and the individual's role (e.g., nurses and administrators will be trained together in the last two modules, but administrators will not be present while the nurses practice use of the OHAT). Initial training will be didactic and hands-on, followed by shadowing and problem-solving. Table 2 (next page) details the training schedule by week, hours, and participant. Since some staff must remain on duty while others are trained, it is expected that the training schedule will be implemented twice per NH at the initiation of the project. CNAs hired during the project will view the MCWB training DVD and receive in-service training by the dental hygienist and dementia specialist during their monthly QI visits (detailed later); in addition, staff-wide "refresher" trainings will be held every three months, coincident with that month's QI meeting. Of note, in terms of the pragmatics of this training for wide-scale adoption, the MCWB program includes three options for on-site training, including one that provides three months of support.

Identifying and training the Oral Care Aide. Supervisors will identify one CNA to work as a dedicated OCA. This individual must: (a) be an experienced staff member; (b) be highly regarded; (c) have worked in the setting for at least one year; (d) most frequently work on the day shift; (e) be rarely absent from work; (f) be expected to remain on staff for the duration of the study; and (g) desire the position of OCA. From our experience, we know that this position will be seen as a "career ladder" which relates to improved self-confidence, retention, and quality care.[80] The OCA will receive training above and beyond that provided to the CNAs, most especially related to: (1) providing mouth care to individuals who are resistive; and (2) working with the nurse supervisor to use the Pneumonia Risk Estimation Scorecard to identify residents at highest risk for pneumonia. However, recognizing that no predictive measures have a 100% positive predictive value, the nurse and OCA will be trained to use the scorecard as advisory, and to select residents for care by the OCA based on who in their estimation requires the care of the dedicated OCA to prevent pneumonia. The nursing staff and OCA will be taught how to complete the scorecard and will do so for all new admissions. At the initiation of the project, however, for purposes of efficiency, they will be provided with copies of the scorecards completed by the research staff (i.e., the baseline data related to pneumonia risk). Of note, use of the MDS data to derive the scorecard score makes pneumonia risk estimation especially pragmatic for NH staff.

Table 2. Number of Hours of Training by Week, Day, and Participant

Participant	Week 1			Week 2		Weeks 3-8
	Day 1	Day 2	Day 3	Day 1	Day 2	Day 1
CNAs (and OCA)	4*+	4*+	4	4	4	4
Nurse supervisors	2*+	2*+	2*#	2*#	2	1
Administrator	2*+	2*+	2*#	2*#	1	PRN

* First hour DVD + With all others # With nurses and administrator

Oral Care Aide duties and salary. The OCA will be trained to focus on providing care to residents at greatest risk for pneumonia based on the Pneumonia Risk Estimation Scorecard and staff recommendations. The OCA will work with the identified residents, developing successful strategies to provide mouth care, for as long as his/her specialized skill is needed; this model is consistent with what has become a preferred practice of consistent staff assignment in NHs.[81] Over time, once success is achieved, the OCA will transition these residents to the care of their assigned CNA, and will teach the CNA the individualized, successful strategies for that resident. This transition will allow the OCA to assume responsibility for the most needy /challenging residents at all times.

For this project, the OCA's salary will be paid by the research grant. Should MCWB prove successful in reducing pneumonia and related hospitalizations, consideration will need to be given to covering the costs of the OCA in a more permanent manner. The Medicare program may benefit the most from reduced hospitalizations in terms of reduced expenditures, and the evaluation of these costs is a key component of this project (see cost-effectiveness later in the proposal).

Quality improvement techniques. The QI techniques include a QI team, regular QI meetings, and Quality Circles. These techniques are modeled after those used in two of our own successful projects conducted in long-term care settings related to falls prevention[82] and antibiotic prescribing reduction.[83] A QI team will be assembled at each intervention NH consisting of the administrator, Director of Nursing (DON), the OCA, and selected other nurses and CNAs, as determined by each NH. The QI team will convene monthly meetings, attended also by the principal investigator, dementia specialist, dental hygienist, and project coordinator. Open discussion will ensue regarding the mouth care program and related barriers and facilitators; also, information specific to implementation and outcomes will be reviewed. This information includes, for example, the frequency of mouth care provision based on logs completed by the CNAs and OCA, information related to resident's oral health status obtained on 20 randomly selected residents at six month intervals throughout the project, and the use and benefit of the scorecard for selecting residents cared for by the OCA. The QI team may choose to establish benchmarks toward which to strive for these and other indicators. Procedures will be put in place so that all CNAs are informed of the success of the QI program on a regular basis, such as through the provision of posted "Mouth Care Briefs" that summarize the information noted above. In addition, Quality Circles facilitated by research staff will be offered to NHs. In Quality Circles, staff within and across settings meet regularly to talk about their experiences and share suggestions. As a component of total quality management, Quality Circles recognize that peer support provides motivation and increases performance.[84-85]

Evaluation: Methods and Measures. This section provides information regarding the data to be collected for evaluating the intervention, and the methods for doing so. To obtain the chart-based data, we will request a waiver from the Institutional Review Board of the University of North Carolina at Chapel Hill permitting limited access to and abstraction of protected health information from resident charts, per 45 CFR 164.512(i)(1)(i).

Pneumonia (rate and incidence). The NH pneumonia rate will be assessed by review of all residents' NH charts for six months prior to the project's inception (for matching purposes) and quarterly throughout the

project (including charts of residents discharged or deceased since the last chart review). Establishing a definitive diagnosis of pneumonia in NH residents is challenging. No accepted clinical criteria exist, in large part due to atypical presentations of pneumonia symptoms among older persons, including subtle clinical manifestations such as falls, incontinence, failure to eat, or acute delirium,[2,86] and also because diagnostic x-rays are not always performed and can be inaccurate.[63] Therefore, we will use a modification of the method of Nicolle and colleagues[87] to assure we capture all cases. Specifically, the existence of any one of the following definitions will be considered to be a pneumonia: (1) X-ray infiltrate and at least one of the following: respiratory rate > 25, reduction in oxygen saturation of at least 3%, new dyspnea, or new cough;[1] (2) clinical diagnosis of pneumonia by a treating physician, nurse practitioner, or physician assistant (a commonly-used research criterion);[87-89] and (3) prescription of an antibiotic for a presumed pneumonia, also a commonly-used research criterion.[73,87,90] A resident whose chart indicates having met any of these definitions will be considered to have had pneumonia. While we do not expect bias in detection across treatment and control sites, we will assess for bias by determining the extent to which each of these definitions was used to identify cases of pneumonia. Finally, to avoid duplicate assignment to pneumonia cases that do not initially respond to therapy, a new case in the same resident will be considered to have occurred only if antibiotic treatment of a prior pneumonia has been discontinued for at least one week, a criterion used in prior research.[91]

Oral health status. Recognizing that oral health status is the mechanism through which MCWB is expected to reduce pneumonia, the oral health of a random sample of 60 residents at each of the 14 NHs will be evaluated at baseline and 24 months by a dental hygienist. Three measures will be used for this purpose, all of which were used in our prior work. The Plaque Index for Long-Term Care (PI-LTC) is a modification of the Simplified Oral Hygiene Index.[92] It is derived by separately scoring the buccal and lingual surfaces of six sextants in the mouth (left, front, and right regions of the upper and lower jaw), resulting in 12 separate observations for residents with a full set of teeth; sextants not containing teeth do not receive a score. Within each sextant, the tooth surface with the worst plaque is scratched using an explorer and assigned a score (0=no plaque or stain present; 1=soft plaque covering not more than one third of the tooth surface or presence of extrinsic stains without other plaque regardless of surface area covered; 2=soft plaque covering between one third and two thirds of the tooth surface; or 3=soft plaque covering more than two thirds of the exposed tooth surface). PI-LTC scores range from 0-3 and are calculated as the sum of the individual sextant scores divided by the number of sextants scored. Separate scores for buccal and lingual surfaces also are calculated. The Gingival Index for Long-Term Care (GI-LTC) is a modification of the Gingival Index.[93-94] Within each sextant, the most inflamed gingival surface is identified, swept using an explorer, and assigned a score (0=no inflammation; 1=mild inflammation, slight change in color, little change in texture; 2=moderate inflammation, glazing, redness, edema, and/or hypertrophy; or 3=severe inflammation, marked redness, edema and/or hypertrophy of the marginal or papillary gingival unit, spontaneous bleeding, congestion, or ulceration). Overall GI-LTC scores range from 0-3 and are the average sextant score. Buccal and lingual surfaces scores also are calculated. The Denture Plaque Index (DPI) [95] is scored by removing the denture, placing it in a bath of disclosing solution for 30 seconds, rinsing it under lukewarm water for 15 seconds, and assigning a score to each of four quadrants (upper and lower, and lingual and buccal) as follows: 0=no plaque, 1=light plaque (1-25% of area covered), 2=moderate plaque (26-50% of area covered), 3=heavy plaque (51-75% of area covered), or 4=very heavy plaque (76-100% of area covered). All three measures are included in Appendix 3.

The dental hygienist conducting the oral health assessments will be blind to the study condition status of the NH (intervention vs. control). He or she will be trained by the dental investigator (Dr. Chen) to assure at least 80% reliability with Dr. Chen's assessment; similar procedures in other studies have achieved reliability ranging from 88-97%.[96] The costs of these assessments are research costs that will not be included in the cost assessment. If the hygienist detects a potential dental emergency (e.g., cancerous growth), he/she will notify Dr. Chen who then will follow-up with the NH supervisor. We expect this situation to be the only one that might constitute an "intervention" in the control sites, but it is expected to occur rarely if at all.

Recruitment and Consent. All residents will be eligible for the assessment of oral health status and will be randomly selected from the NH roster. Consent will be obtained from the resident or his/her legally authorized representative (LAR) if the resident is unable to consent based on the administration of a reliable and widely used six-item cognitive screening instrument.[97] Using IRB-approved procedures we have used numerous times

[1] While the finding of a new infiltrate on chest x-ray is sometimes considered highly specific as a diagnostic criterion for pneumonia, inclusion of a pneumonia-related symptom in addition to the x-ray finding is necessary because of the poor reliability (r = 0.5) of radiologists' interpretations of portable x-rays obtained in NHs.[64]

in past studies, a research assistant will approach residents and explain the study and all issues related to informed consent (e.g., the right to stop participation at any time). LARs will receive a letter followed by a telephone call for those who are willing to learn more about the study; they will be asked to provide consent for the resident's participation, and assent will be obtained from the resident. Consent will indicate the intention to contact a physician to prescribe an antibiotic prior to the assessment if the resident has a condition that may put him/her at risk of infective endocarditis (e.g., artificial heart valve) which was applicable to fewer than 2% in our prior study).[101] Recruitment will continue in random order until the oral status of 60 residents has been evaluated.

Quality improvement oral health status. For QI purposes, the oral health of 20 randomly selected residents in intervention sites only will be assessed by the hygienist every six months using procedures identical to those described above. It is important to restate that the nursing supervisor also will be assessing the condition of residents' teeth and gums with the OHAT.

Hospitalization, mortality, and facility-level covariates. As noted earlier, NH charts will be reviewed for the period six months prior to the project's inception and quarterly throughout the project to determine pneumonia rates and incidence. At the same time, data will be collected related to resident discharge (including date), hospitalization (including date and diagnosis, obtained from the hospital discharge summary), and mortality (including date), and whether the resident had documentation of having received the pneumococcal vaccine (MDS item O03). If a resident dies while in the hospital, the reason for admission will be obtained.

NH level-data will be collected to assess the success of randomization and, to a limited degree, for use as covariates in secondary analyses. These data, which are based on health inspections and the MDS, are publically available at https://data.medicare.gov; they are updated as new annual reports are received, and so remain timely. They include information on NH demographics (e.g., ownership), case-mix (e.g., the percent of short-stay residents, with weight loss, pressure ulcers, assessed/given the pneumococcal vaccine), staffing (e.g., registered nurse and CNA hours/resident day), and quality measures suitable for risk adjustment. In addition, an interview will be conducted with the administrative staff in all NHs to learn about standard mouth care provided, including details of dental and dental hygienist services availability (see Appendix 3).

Implementation and sustainability of Mouth Care Without a Battle. To better understand the implementation and sustainability of MCWB, data will be collected regarding staff self-efficacy, frequency of mouth care and related supervision, use of mouth care products, barriers and facilitators to implementation, and cost.

Staff self-efficacy to provide mouth care. Our measure of staff self-efficacy was developed from other sources[98-100] and used successfully in our prior work.[101] It will be administered to all CNAs (age 21 and older) in the intervention sites at baseline and 24 months, and to all CNAs (age 21 and older) in the control sites at 24 months (to avoid a possible Hawthorne effect from pre-administration). In the intervention sites, the 24-month measure will include a retrospective pre-test that asks respondents to reflect on their initial self-efficacy after having learned what they might not have known that they did not know previously. This technique is useful to learn reflections about the perceived effectiveness of a program[102] and was used in our prior work. The self-efficacy measure has 35 items (Cronbach's alpha α=0.74-0.92), scored 1 (strongly disagree) to 4 (strongly agree). Sample items include "I am familiar with the practical procedures to do this job" and "I know ways to successfully provide mouth care to residents who hit or scream", both of which evidenced significant improvement in our prior work. (See Appendix 3.) Arrangements will be made with the NH administrator for the research assistant to obtain staff consent, distribute the questionnaire, and oversee its completion, during shift change.

Frequency of mouth care will be documented on a simple log developed for our prior work, on which the OCA and CNA record daily, for each resident, whether mouth care occurred. If mouth care did not occur, a reason is documented. In addition, the OCA will document successful strategies used with residents, which can then be shared with the CNA if/when the care of that resident reverts to the care of the CNA. This information will be reviewed with staff during QI meetings and also used for analysis. (See Appendix 3.)

Time spent in nurse supervisory activities (assessing residents, working with the CNAs and OCA related to mouth care) will be logged on a sheet developed for this purpose. This information will be reviewed with staff during QI meetings and also used for analysis. (See Appendix 3.)

Use of mouth care products will be documented whenever a CNA or OCA indicates on the mouth care log that mouth care was provided. Product use also will be monitored by the project office every three months, based upon the frequency with which supplies must be replaced (with use determined on a per resident level). This information will be reviewed with staff during QI meetings and also used for analysis.

Barriers, facilitators, and sustainability (e.g., time demands, ready access to products) will be discussed and documented at every QI meeting. In addition, interviews asking about each of the topics identified during QI meetings will be conducted at 24 months with members of the QI team, other supervising nurses, the administrator (if not included), and a random sample of five CNAs in each NH. Interviews will include open-ended questions and Likert scales to rate frequency/importance/intensity.

Intervention costs. In the intervention NHs, costs measured over the two year study period will include the salary of the OCA and average salaries of the CNAs and nurses; staff time for MCWB training, supervision, and QI meetings; time spent providing mouth care; and product costs. In addition, we will track staff turnover. Only costs pertaining to implementing and maintaining the intervention will be included in the analysis; research costs will be excluded. We will measure training (initially and rehires) based on the receipt of continuing education credit which is arranged through the project office, as well as time logs maintained by Ms. Poole and Dr. Sloane. The nurse supervisors' logs will include an area to record time spent. The number of staff who attend QI meetings, as well as the length of the meetings and time required for preparation and follow-up, will be compiled by the project coordinator. An estimate of the amount of time spent providing mouth care will be based on observations conducted by the research assistant (at baseline and quarterly time points) of mouth care provided by two randomly selected CNAs to approximately 12 residents over two mornings. The research assistant will shadow these staff, documenting time spent per resident and completeness of mouth care based on the MCWB protocol. To avoid an "intervention effect" from this activity (especially at baseline), the research assistant will record time for all personal care tasks provided at the same time (e.g., dressing).

Power. Our estimate of statistical power is focused on Aim 1a, the primary analysis. For this, a base (pre-intervention) pneumonia rate of 2 cases per 1,000 resident days was assumed. Troy et al.[103] report that NH rates vary from 0.4 to 4.4, while Muder[104] reports a range of 0.3 to 2.5. We assume a rate in the upper part of the range because the selected NHs have above average hospitalization rates and other characteristics associated with higher pneumonia incidence. To adjust for variance inflation due to both variation in pneumonia risk among residents and variation among NHs, an overdispersion factor of 3 was assumed.

With an average of 102 residents per site over the course of the observation period (based on current censuses at the prospective sites) there would be a total of 74,460 resident-days observed per site, resulting in an expected 149 pneumonia cases per control site over two years. The number of unique residents observed per site is expected to be around 210 for an overall total of about 2940 residents (considering replacement over time), with an average observation period of approximately one year. Given these assumptions, there would be 80% power to detect a reduction of 19% in the base rate to 121 cases per intervention site with an alpha = .05 one-tailed test. If the base rate is as low as 1.5 cases per 1000 resident-days or the overdispersion parameter one-third higher, there would still be power to detect a reduction of 25% or more. Even the 25% reduction is substantially less than the nearly 40% reduction reported by Yoneyama[20] for an oral care intervention and thus seems achievable. While this power calculation is based on large sample distributions for an unmatched analysis, it should reasonably approximate the power for the permutation test of the paired t-statistic in Aim 1a.

Because they are intended only to be either exploratory and hypothesis-generating or to provide information about why an intervention effect was (or was not) achieved, formal power analysis for the sensitivity analysis related to Aim 1a and other secondary analyses were not performed.

Analyses. The evaluation of MCWB relates to the incidence of pneumonia (Aim 1, overall and in relation to pneumonia risk and oral health status), implementation and sustainability (Aim 2) and secondarily, if pneumonia incidence is reduced, to hospitalization and mortality (Aim 3).

Aim 1a. Compare the incidence of pneumonia in the mouth care NHs to the incidence in control NHs. For the primary analysis, we will compare intervention and control sites using a permutation test based on a paired t-statistic to test whether the mean difference in log pneumonia incidence rates between control and intervention NHs within matched pairs is greater than zero (indicating an intervention effect to reduce rates). The pneumonia rate for each NH will be computed as the total number of cases of pneumonia divided by the total number of resident-days of exposure. The null permutation distribution of the paired t-statistic results from the $2^7 = 128$ possible sign inversions of the 7 differences in log rates. The permutation test accounts for the fact that NHs were randomized within NH pairs and that there are 128 equally likely ways that intervention assignments could have occurred during randomization. The rank of the observed paired t-statistic among all 128 possible statistics provides the p-value; a one-sided test rejects at the 0.05 level if the observed statistic falls among the largest 0.05 x 128 = 6 values. The permutation test ameliorates the impact of outlying log rate

ratio values and the heterogeneity of their variances resulting from varying numbers of residents and follow-up times within homes. To the extent that matched sites are indeed similar, this approach will increase power to detect an intervention effect relative to an unmatched analysis.[105] Also, as done in another project,[106] a sensitivity analysis will be conducted to assess the impact on the estimated intervention effect of possible random baseline imbalances in resident-level covariates (including pneumonia risk) between intervention and control homes. This analysis will be carried out by first computing a risk-adjusted log pneumonia rate for each NH via a negative binomial model with NH-specific fixed effects intercepts, and then applying a permutation test to the within-pair differences in NH-level residuals computed as observed log rate minus expected log rate.[107] A bootstrap confidence interval using the quantile method will be constructed for the mean difference in log rate of pneumonia within NH pairs; 90% confidence intervals will be reported for the incidence rate ratio by exponentiating the obtained bounds, corresponding to one-sided tests at the p=0.05 level.

Aim 1b. Compare the case-adjusted incidence of pneumonia in mouth care NHs to the case-adjusted incidence in control homes, and evaluate whether the intervention effects for pneumonia incidence are modified by resident-level risk factors for pneumonia. For the secondary analysis for Aim 1b, a generalized linear mixed model with log link, Poisson variance, random effects for sites, caregivers, and residents, and fixed effects for resident-level characteristics, will be employed. A limited number of NH characteristics will be included in the model if randomization resulted in their imbalance for intervention versus control conditions. Interaction terms involving resident risk factors with a binary indicator for OCA involvement will assess effect modification, specifically, identification of subgroups of residents for whom the intervention may have a differential impact relative to other subgroups.

Aim 1c. Examine the relationship between the overall incidence of pneumonia and change in average oral health status, as measured by the plaque and gingival indices of cross-sectional random samples of 60 residents per NH at baseline and at the end of the study. For the primary part of the secondary analysis for Aim 1c (testing for the effect of the PI-LTC, GI-LTC, and DPI on the incidence of pneumonia), the average value per site of each oral health measure will be calculated for both baseline and 24 month measures, and the difference between them determined. The value for each measure, representing overall change in oral health for the site during the intervention period, will be correlated with the pneumonia rate for each site during the intervention period, and a 95% confidence interval for the correlation will be computed. The values also will be plotted for a visual representation of the relationship. In a subsequent exploratory analysis, we will perform regression analysis with resident-level data to additionally assess the population-average effects of the intervention on change in oral health status adjusting for resident characteristics. In particular, we will apply linear mixed models with site, caregiver, and resident as random effects and fixed effects for period (baseline or follow-up), intervention status with care from an OCA, intervention status with care from a CNA, and interaction terms for each of the intervention statuses by period. This strategy will permit testing of the overall effect of the intervention as well as testing of the individual contrasts of OCA care and CNA intervention site care with control site care and with each other. To obtain the most interpretable parameters, period and intervention status variables will be coded as dummy variables with values of 0 (baseline, control) or 1 (follow-up, intervention).

Finally, we will conduct a supplemental analysis that tests for a dose-response relationship between the amount of oral care delivered and the PI-LTC, GI-LTC, and DPI for those residents in intervention sites receiving an oral exam at follow-up (acknowledging that under the repeated cross-sectional design, few residents will receive both baseline and follow-up exams). For that analysis, an additional linear mixed model will be constructed that has the same random effects as above and fixed effects for dose of treatment received from OCAs and dose of treatment received from CNAs. The dose measures for OCA and CNA will each be derived from QI/fidelity data and coded as the number of days that oral care was delivered. The analysis will be adjusted for resident characteristics believed to be associated with oral health as well as the facility-level average of the baseline outcome score (PI-LTC, GI-LTC, and DPI). Due to selection effects (i.e., OCAs will be more likely assigned to residents with worse oral health than CNAs), comparisons between OCAs and CNAs will not be made, but rather assessment will be focused on dose effects within OCA or CNA. A simultaneous analysis of OCA and CNA effects will be performed because residents who receive care from the OCA may also receive care from CNAs.

Aim 2. Implementation and sustainability. To assess change in CNA and OCA self-efficacy, tests of contrasts for effects representing temporal change in scores within OCAs and CNAs, respectively, at intervention sites, and post-intervention differences between intervention OCAs, intervention CNAs, and control site CNAs, will

be conducted using linear mixed models with random effects for site and staff person, and fixed effects consisting of four indicator variables to represent the five group-period combinations (OCA-baseline, CNA-baseline, Control-followup, OCA-followup, CNA-followup). Both baseline pretest and retrospective pretest values will be considered, in separate analyses, as representative of the pretest value. For frequency of mouth care and use of mouth care products, three measures of the implementation of the oral care protocol in intervention sites will be used: first, whether any part of prescribed care was provided (frequency: yes/no), second, the extent to which prescribed care was performed (dose) and, third, the quantity of care products used. Individual sites will be evaluated on all three measures for facility-level achievement at each measurement point and for the trend over time. Comparisons will be made between sites with graphs, and differences tested using repeated measures multivariate analysis of variance. Each site's results from these analyses will be reported to that site for use in QI.

Analyses related to barriers and facilitators will be primarily qualitative in nature, but also will derive ranked scores of frequency/importance/intensity. For qualitative data, using standard analytic techniques that we have used in other projects, three researchers (SZ, PS, LC) will review notes from the first QI meeting in each NH and, using open coding (i.e., breaking the data into the smallest unit), will develop manifest and latent codes (e.g., time, products, supervision, satisfaction, family). They will meet to discuss discrepancies in coding, reach consensus, and then apply the resulting codes to the materials from the other QI meetings and summative interviews. Two individuals will code every tenth document and if discrepancies are noted they will be resolved before coding continues. Then, the coded material will be examined to discern the relationship between codes to identify broader themes (e.g., family satisfaction overriding time constraints).[108]

Finally, estimation of total facility program costs will be done separately according to implementation and sustainability. Implementation costs will be viewed as "sunk costs" while maintenance costs will be most relevant for the economic evaluation of the cost-effectiveness of the program if the intervention is found to reduce the rate of pneumonia. Research costs will not be included in either calculation. Implementation costs will consist of training costs by using time estimates for all NH staff involved in training; these estimates will be adjusted for NH size. We will construct an estimate of annual oral health care costs by considering product cost, time spent by the OCA and CNAs providing mouth care (described earlier), training of new staff hired after initial implementation, and estimates of management and administration costs provided by supervisors.

One important issue for widespread program implementation after the trial pertains to coverage of the salary for the OCA and training costs. In practice, the NH would be responsible for covering such costs unless another source were identified. Beneficiaries of the program include NH residents (improved care and avoidance of pneumonia and hospitalization) and their families; the NH itself (improved quality ratings on public reports and resident and family satisfaction, both of which could result in higher occupancy); and the Medicare program in terms of reduced hospital costs from avoided hospitalizations. Medicare is actively interested in interventions to reduce hospitalizations from NHs, so evidence from this study will be helpful to broader considerations of appropriate reimbursement for improvements in quality that reduce hospitalization.

Aim 3a. Compare differences in hospitalization and mortality rates (pneumonia and all cause) from baseline through two years for mouth care and control NHs. For analysis of Aim 3a, log hospitalization and mortality rates will be compared between intervention and control sites using negative binomial regression models with random intercepts for NHs to account for between-facility heterogeneity in rates. Resident-level characteristics will be included as fixed effects to provide case-adjustment, and NH characteristics that were not balanced in the randomization will be included. Exponentiation of the regression coefficient for intervention effect will provide an estimate of the incidence rate ratios for hospitalization and mortality rates, respectively, for which 95% confidence intervals will be reported. We will estimate hospitalization costs by tracking the total days hospitalized and the diagnosis related to the hospitalization. We will use estimates of Medicare payments for the conditions hospitalized from the Healthcare Cost and Utilization Project.[109]

Aim 3b. Estimate the cost per hospitalization avoided attributable to the mouth care program. Analyses related to Aim 3b will assess the potential for cost-savings of the mouth care program using standard methods for economic evaluation.[110-112] We will estimate cost-effectiveness or net benefits using estimated program costs and hospitalization costs. We will first assess the likelihood of cost-savings. Assuming that the chance of the program being cost-saving will be less than 100%, the main measure of effect will be the number of hospitalizations avoided. The methods will be very similar to our recent studies of the cost-effectiveness of an oral health care program for young children eligible for Medicaid[113] and a preventive intervention for asthma.[114]

One complication for models of the outcomes of hospitalizations and spending is that if observation periods vary due to the death of some subjects and the loss to follow-up of others, and if covariates such as disease prevalence affecting resource use also affect survival, biased estimates of resource use trajectories can arise. To address this problem, we will use recently developed methods that allow treatment effects on resource use to be decomposed into effects attributable to survival differences versus effects caused by differing intensity of utilization conditional upon survival.[115] A three-part estimator can be used to provide robust estimates of differences in hospital use and spending over time when some observations are censored due to death or different follow-up periods. The estimator also decomposes the differences into effects attributable to survival differences versus treatment-related intensity of resource use.

Cost-effectiveness analysis generally involves the calculation of an incremental cost effectiveness ratio (ICER), which gives the cost per additional unit of outcome or effect as shown in this formula:

$$ICER = \frac{Cost_{InterventionGroup} - Cost_{ControlGroup}}{Effect_{InterventionGroup} - Effect_{ControlGroup}}$$

Costs in the numerator include estimated costs of hospitalizations for both groups; the costs for the intervention group also include program costs. Since hospitalization is an undesirable outcome, the denominator will be estimated by subtracting the hospitalization rate for the intervention group from the rate for the control group. Positive values of the ICER generally reflect situations in which an intervention leads to better outcomes at increased costs. In some cases, the ratio can be negative because outcomes are better and costs are lower for the intervention group, in which case an intervention is cost-saving. Both the numerator and denominator are estimated with uncertainty and may have correlations between them; however, methods for dealing with such uncertainty and correlations have been developed.[116-117] These methods use bootstrapping or Monte Carlo simulations to characterize the likelihood of an intervention being cost-saving, use graphic representations such as cost-effectiveness acceptability curves to allow depiction of the likelihood that an intervention is cost-effective relative to the amount someone (e.g., a policy maker) would be willing to pay per unit improvement in outcome. As noted earlier, the sample size for the trial is based on the power needed to determine a statistically significant improvement in the health outcome (i.e., a reduction in the incidence of pneumonia, as is standard for many clinical trials); the sample is small relative to that which would be needed to get a precise estimate of the ICER. However, methods accounting for uncertainty in the estimation of the ICER will enable an estimate of the likelihood that the intervention will be cost-savings.

Scorecard validation. The primary purpose of the Pneumonia Risk Estimation Scorecard is to provide a general estimation of risk for use by the NH staff and in analyses. In addition, we will use data from control site residents to calculate the area under the ROC curve as an overall measure of diagnostic accuracy, and will select a cutpoint to test for the positive predictive value, sensitivity, and specificity of the scorecard.

Data and Safety Monitoring Board. With approval of the program official, a Data and Safety Monitoring Board (DSMB) composed of individuals with expertise in mouth care provision in NHs, pneumonia and mortality in NHs, pneumonia and dementia in NHs, and biostatistics, will meet at least annually to review the protocol and data as related to safety, study conduct, and progress. Material related to safety that likely will be reviewed includes dental emergencies (e.g., acute infection of the teeth or gums, cancerous growth), non-emergencies that indicate a need for dental referral (e.g., loose dentures, pain), and others. The investigators will work with the DSMB at the initiation of the study to identify the materials they wish to review and the protocol for obtaining those materials, as well as other matters related to their charge.

Final Considerations. In smaller NHs, OCAs will provide care for a greater proportion of residents than in larger NHs; however, because the intervention is facility-wide and provided by all CNAs, and because NH pairs are matched by size, concern about this difference is tempered. Also, it is possible that control NHs may evidence a reduction in hospitalizations due to more attention to falls; however, baseline falls rates will be available as a point of comparison, and analyses can still determine cost per desirable outcome.

Timeline. The timeline for this five year study is provided in Figure 4.

Figure 4. Timeline.	Year 01				Year 02				Year 03				Year 04				Year 05			
Finalize protocol/measures, randomize site, order supplies, hire hygienist																				
Convene Data and Safety Monitoring Board (DSMB)																				
Train/calibrate data collectors and dental hygienist																				
Train on Mouth Care Without a Battle (MCWB); rolling onset																				
Maintain MCWB through QI techniques; provide falls data to control NHs																				
Conduct oral health assessments, collect outcome and other data																				
Enter and clean data; create datasets																				
Collect sustainability data; analyze data (*preliminary analysis for DSMB)				*				*				*								
Prepare manuscripts, make presentations; deliver MCWB to control sites																				
