# Supplementary material for: Effectiveness of a Mouth Care Program Provided by Nursing Home Staff vs Standard Care on Reducing Pneumonia Incidence: A Cluster Randomized Trial
Source: JAMA Netw Open. 2020 Jun 19;3(6):e204321. doi: 10.1001/jamanetworkopen.2020.4321 (PMC7305523; doi:10.1001/jamanetworkopen.2020.4321)
Supplement: Supplement 2. — eAppendix. Supplementary Methods and Results eTable 1. Baseline Nursing Home Characteristics and Characteristics of Residents Participating in Oral Hygiene Assessments eTable 2. Mouth Care Provision Over Two Years Based on Mouth Care Logs for Residents in Mouth Care Without a Battle Nursing Homes eTable 3. Oral Hygiene of Repeated Cross-sectional Samples of Residents in 14 Nursing Homes During 2 Years eTable 4. Hierarchical Linear Mixed Model Estimates of Effects on Plaque and Gingival Oral Hygiene eTable 5. Two-Level Linear Mixed Model Estimates of Effects on Denture Plaque eFigure. Plaque, Gingival, and Denture Plaque Index Scores, Baseline Through 24 Months [file jamanetwopen-3-e204321-s002.pdf]

Supplementary Online Content

Zimmerman S, Sloane PD, Ward K, et al. Effectiveness of a mouth care program provided by nursing home staff vs standard care on reducing pneumonia incidence: a cluster randomized trial. *JAMA Netw Open*. 2020;3(6):e204321. doi:10.1001/jamanetworkopen.2020.4321

eAppendix. Supplementary Methods and Results
eTable 1. Baseline Nursing Home Characteristics and Characteristics of Residents Participating in Oral Hygiene Assessments
eTable 2. Mouth Care Provision Over Two Years Based on Mouth Care Logs for Residents in Mouth Care Without a Battle Nursing Homes
eTable 3. Oral Hygiene of Repeated Cross-sectional Samples of Residents in 14 Nursing Homes During 2 Years
eTable 4. Hierarchical Linear Mixed Model Estimates of Effects on Plaque and Gingival Oral Hygiene
eTable 5. Two-Level Linear Mixed Model Estimates of Effects on Denture Plaque
eFigure. Plaque, Gingival, and Denture Plaque Index Scores, Baseline Through 24 Months

This supplementary material has been provided by the authors to give readers additional information about their work.

© 2020 Zimmerman S et al. *JAMA Network Open.*

eAppendix. Supplementary Methods and Results

Staff who provide daily mouth care using *Mouth Care Without a Battle (MCWB)* can improve residents' oral hygiene, as discussed in previous work.[1,2]

Because change in oral health status is the mechanism through which *MCWB* is expected to reduce pneumonia, assessing the change in residents' oral health is an indicator of fidelity and sustainability to *MCWB*.

Fidelity and sustainability to *MCWB* was measured using two methods: daily mouth care logs (a measure of care provision) completed by certified nursing assistants, and observations of oral hygiene.

METHODS AND MEASURES

Daily mouth care logs. Frequency of mouth care was documented on a simple log developed in our prior work, on which the Oral Care Aide (OCA) and Certified Nursing Assistant (CNA) recorded daily, for each resident, whether mouth care occurred. If mouth care occurred, the OCA or CNA would record the following information: date care was provided; type of product used to brush/cleanse the resident's teeth or gums; use of interdental brush; use of fluoride paste; and if applicable, whether the denture was cleaned. In addition, the log included a section where the OCA or CNA could write notes about mouth condition, challenges to providing care, helpful strategies, and the reason care was not provided.

Oral hygiene. Oral hygiene was assessed by a dental hygienist and collected for a random sample of residents in each of the seven *MCWB* nursing homes (NHs) at baseline and at 4, 12, 20, and 24 months later. Up to 60 residents were assessed at baseline and 24 months, and up to 20 residents were assessed at 4, 12, and 20 months, in each NH. Oral hygiene of selected tooth surfaces was assessed using the Plaque Index for Long-Term Care (PI-LTC), the Gingival Index for Long-Term Care (GI-LTC), and the Denture Plaque Index (DPI); for all indices, higher scores indicate worse oral hygiene.

- PI-LTC: a modified version of the Debris Index of the Simplified Oral Hygiene Index[3] scored from 0 (no plaque or stain) to 3 (soft plaque covering more than two-thirds of tooth surface).
- GI-LTC: a modified version of the Gingival Index[4] scored from 0 (no inflammation) to 4 (severe inflammation).
    - For the PI-LTC and GI-LTC, a score was assigned for the worst buccal or lingual surface within each of the 3 maxillary and 3 mandibular sextants (i.e., most plaque, most inflammation). These individual scores were used to create mean maxillary, mandibular and overall summary scores.
- Denture Plaque Index (DPI):[5] used even for partial dentures, and obtained by immersing dentures in a disclosing solution for 30 seconds, rinsing off excess dye for 15 seconds, and assigning a score of 0 (no plaque) to 4 (very heavy plaque covering >75% of the area).
    - Scores were assessed for each maxillary and mandibular facial and basal quadrant. In addition, these individual scores were used to create a mean summary score.

Analysis. Oral hygiene outcomes were first examined by calculating observed mean scores across the five time periods for the *MCWB* NHs and the two time periods for the control NHs for all oral hygiene individual and aggregate measures. To assess change in oral hygiene over 24 months, a linear mixed effects model was used to handle the repeated cross-sectional samples. The model specified the oral hygiene measures as the outcomes, independent random intercepts for residents and sites, a fixed effect for categorical time (baseline = 0; 4 months = 1; 12 months = 2; 20 months = 3; 24 months = 4), a fixed effect for arm (*MCWB* = 0, control = 1), and a 24 months X control fixed effect interaction term. Under this framework, the interaction term provides the difference-in-differences estimates to compare *MCWB* versus control with respect to population-averaged 24 month change in oral hygiene status. A positive coefficient and significant p value (<.05) indicate positive improvements to residents' oral hygiene due to *MCWB* participation relative to baseline and control.

RESULTS

Sample. Table 1 describes the NHs participating in the project and the residents participating in oral hygiene assessments. NH characteristics were similar across all variables compared. Residents exhibited some differences across arms; MCWB residents were younger, less likely to be female, more likely to be other than White or African American, were more likely to have Alzheimer's disease or dementia, were more likely to have swallowing issues, varied in eating dependency, were less likely to have teeth, and were more likely to have had a flu vaccine.

© 2020 Zimmerman S et al. *JAMA Network Open.*

Daily mouth care logs. Table 2 describes mouth care provision based on the daily mouth care logs. In the first year, 832 residents (98.9% of those for whom logs were completed) successfully received mouth care over 77,310 episodes of care (32 times per 100 resident days; 9.6 days per resident month/30 days). In the second year, the corresponding numbers were 566 residents (97.6%) over 56,115 episodes (26 times per 100 resident days; 7.8 days per resident month/30 days). Overall, the probability of receiving mouth care was 0.29 times per resident day over the entire two years. The proportion of residents who refused mouth care decreased from 3.8% of resident days to 1.8% of resident days from the first to the second year. When mouth care was provided, the most frequently used product was chlorhexidine (76.5% and 38.6% of resident days in the first and second year, respectively). Interdental brushes were used 34.1% and 28% of resident days.

Oral hygiene. Table 3 displays detailed data for plaque, gingiva and denture plaque over five observations (*MCWB* homes) and two observations (control homes), and Tables 4 and 5 display the mixed model effects on plaque, gingiva, and denture plaque. As shown in Table 4, change from baseline peaked at 12 months, and decreased at 20 and 24 months; for example, plaque scores across all sextants (column 1) indicated a .78, .96, .55, and .45 decrease (improvement) from baseline at 4, 12, 20, and 24 months respectively (all $p<.001$). The same trend is evident for the gingival index. Also, as shown in the "interaction" row, over 24 months and relative to the intervention, the control group had a .35 increase in the plaque score, and a .46 increase in the gingival score, indicating worse oral hygiene ($p<.001$). Similar results are shown for denture plaque in Table 5.

Figure 1 displays the "all sextants/surfaces" data graphically. All scores were similar at baseline (1.73 vs. 1.65, 1.60 vs. 1.47, and 2.25 vs. 2.14, for plaque, gingiva, and denture plaque, respectively; see Table 3). Scores at 24 months were improved for *MCWB* homes compared to control homes (1.20 vs. 1.50, 1.12 vs. 1.45, and 1.64 vs. 1.93, for plaque, gingiva, and denture plaque, respectively).

DISCUSSION

Mouth care was provided less often in Year 2 compared to Year 1, and mouth care in Year 2 less often used recommended products. Relatedly, oral hygiene was most improved at 12 months, with the improvement lessening through 24 months. Despite less improvement over time, oral hygiene was consistently significantly better in *MCWB* homes than control homes.

One limitation to these analyses is that the data regarding care provision are restricted to those recorded by NH staff, and the amount of missing data cannot be quantified.

REFERENCES

1. Sloane PD, Zimmerman S, Chen X, et al. Effect of a person-centered mouth care intervention on care processes and outcomes in three nursing homes. J Am Geriatr Soc. 2013;61(7):1158-1163.
2. Weintraub JA, Zimmerman S, Ward K, et al. Improving nursing home residents' oral hygiene: results of a cluster randomized intervention trial. J Am Med Dir Assoc. 2018;19(12):1086-1091.
3. Greene JG, Vermillion JR. The simplified oral hygiene index. J Am Dent Assoc. 1964;68:7-13.
4. Lobene RR, Weatherford T, Ross NM, et al. A modified gingival index for use in clinical trials. Clin Prev Cent. 1986;8:3-6.
5. Augsburger RH, Elahi JM. Evaluation of seven proprietary denture cleansers. J Prosthet Dent. 1982;47:356-359.

© 2020 Zimmerman S et al. *JAMA Network Open.*

eTable 1. Baseline Nursing Home Characteristics and Characteristics of Residents Participating in Oral Hygiene Assessments

Characteristics	Mouth Care Homes<br>N (%) or Mean (SD)	Control Homes<br>N (%) or Mean (SD)
**Nursing Home Characteristics**	**7 (50.0)**	**7 (50.0)**
Nursing home		
For profit	6 (85.7)	5 (71.4)
Years in operation	29.1 (8.8)	25.3 (12.4)
Number of licensed beds	105.7 (26.7)	104.3 (25.1)
Percent private rooms	35.0 (35.6)	25.1 (20.2)
Resident case-mix (percent)		
Dementia diagnosis	63.4 (11.6)	56.6 (14.3)
Requires staff attention for behavior	21.4 (19.1)	7.1 (4.9)
Medicaid coverage	61.0 (25.7)	62.1 (15.0)
Medicare coverage	23.7 (12.1)	20.4 (16.8)
Nursing home star rating [a]	3.9 (1.3)	3.6 (1.5)
Staffing		
Registered and licensed nurse minutes per resident day	104.9 (34.0)	112.6 (48.5)
Certified nursing assistant minutes per resident day	158.9 (64.8)	145.6 (27.4)
Oral health services		
On site dentist visits	5 (71.4)	2 (28.6)
On site hygienist visits	2 (28.6)	0 (0.0)
**Resident Characteristics**	**402 (52.8)**	**359 (47.2)**
Demographic characteristics		
Age at baseline	78.9 (12.8)	80.7 (12.1)
Female	241 (65.5)	245 (75.2)
Race/Ethnicity		
White	229 (62.7)	215 (67.2)
African American	102 (28.0)	97 (30.3)
Other	34 (9.3)	8 (2.5)
Health conditions/care		
Chronic renal disease	49 (13.4)	28 (8.6)
Alzheimer’s/dementia	199 (57.2)	136 (42.0)
Malnutrition	10 (2.7)	5 (1.5)
Asthma/COPD	70 (19.1)	63 (19.3)
Recent weight loss	23 (6.4)	18 (5.6)
Swallowing issues	18 (5.7)	6 (1.9)
Feeding tube	15 (4.1)	14 (4.3)
Hospice care	13 (3.6)	15 (4.6)
Dental issues		
No teeth or fragments	33 (9.0)	15 (4.6)
Likely cavities	17 (4.6)	14 (4.3)
Other dental issues	6 (1.9)	6 (1.9)
Medications/vaccinations		
Antibiotic (at baseline)	60 (16.4)	53 (16.3)
Flu vaccine this year	230 (67.7)	157 (52.3)
Pneumococcal vaccine up to date	200 (55.7)	173 (53.2)
Eating dependency		
Independent eating	103 (28.2)	65 (20.0)
Supervised eating	149 (40.8)	161 (49.5)
Limited assistance	22 (6.0)	33 (10.2)
Extensive assistance	70 (19.2)	40 (12.3)
Total dependence	21 (5.8)	26 (8.0)

© 2020 Zimmerman S et al. *JAMA Network Open.*

[a] Nursing home star rating is the Centers for Medicare & Medicaid Services five-star rating scale, which ranges from 1 (low) to 5 (high).

© 2020 Zimmerman S et al. *JAMA Network Open.*

eTable 2. Mouth Care Provision Over Two Years Based on Mouth Care Logs for Residents in Mouth Care Without a Battle Nursing Homes

**Mouth care received**	**Months 1-12** N (%)	**Months 13-24** N (%)
Number of residents for whom logs were completed	841	580
Number of residents receiving mouth care	832 (98.9)	566 (97.6)
Number of times mouth care was attempted (% of resident days)[a]	86,335 (36.0)	59,950 (27.6)
Number of times mouth care was received (% of resident days)[a]	77,310 (32.2)	56,115 (25.8)
Number of times mouth care was refused (% of resident days)[a]	9,025 (3.8)	3,835 (1.8)
**Of times mouth care received, times receiving…**		
Chlorhexidine	59,113 (76.5)	21,685 (38.6)
Biotene or Listerine Total Care Zero	10,034 (13.0)	16,642 (29.7)
Toothpaste	6,299 (8.1)	5,996 (10.7)
Interdental brush	26,352 (34.1)	15,716 (28.0)
Fluoride paste (prescription)	33,215 (43.0)	17,264 (30.8)
Fluoride paste (non-prescription)	3,967 (5.1)	6,083 (10.8)

[a] Denominator for Months 1-12 is 239,805 resident days and for Months 13-24 is 217,560 resident days.

© 2020 Zimmerman S et al. *JAMA Network Open.*

eTable 3. Oral Hygiene of Repeated Cross-sectional Samples of Residents in 14 Nursing Homes During 2 Years (N = 761)

Oral Hygiene Index	Mouth Care Arm (n = 402)					Control Arm (n = 359)	
	**Baseline** n = 265 Mean (SD)	**4 months** n = 161 Mean (SD)	**12 months** n = 161 Mean (SD)	**20 months** n = 129 Mean (SD)	**24 months** n = 236 Mean (SD)	**Baseline** n = 249 Mean (SD)	**24 months** n = 208 Mean (SD)
**Plaque Index for Long-Term Care (0.0–3.0)**							
All sextants (n = 108-211)	1.73 (.75)	.99 (.74)	.80 (.76)	1.14 (.86)	1.20 (.81)	1.65 (.80)	1.50 (.85)
Maxillary (n = 87-174)	1.59 (.80)	.74 (.64)	.65 (.71)	.99 (.86)	.99 (.82)	1.45 (.80)	1.33 (.86)
Mandibular (n = 104-207)	1.82 (.76)	1.09 (.79)	.87 (.80)	1.22 (.90)	1.28 (.83)	1.76 (.82)	1.61 (.87)
**Gingival Index for Long-Term Care (0.0–4.0)**							
All sextants (n = 108-208)	1.60 (.84)	.87 (.81)	.68 (.94)	.99 (.95)	1.12 (.99)	1.47 (.88)	1.45 (1.07)
Maxillary (n = 87-171)	1.49 (.93)	.75 (.81)	.59 (.87)	.90 (.93)	.95 (.99)	1.33 (.91)	1.31 (1.07)
Mandibular (n = 104-205)	1.68 (.84)	.94 (.85)	.75 (.97)	1.08 (1.02)	1.20 (1.04)	1.56 (.90)	1.52 (1.10)
**Denture Plaque Index (0.0–4.0)**							
All surfaces (n = 31-91)	2.25 (1.22)	1.13 (.99)	1.01 (1.14)	1.06 (.99)	1.64 (1.12)	2.14 (1.20)	1.93 (1.18)
Maxillary facial (n = 31-89)	2.31 (1.30)	1.00 (1.04)	1.07 (1.18)	.89 (.89)	1.47 (1.16)	2.06 (1.23)	1.71 (1.24)
Maxillary basal (n = 31-89)	2.31 (1.35)	1.43 (1.13)	1.06 (1.19)	1.34 (1.29)	1.85 (1.27)	2.40 (1.31)	2.39 (1.36)
Mandibular facial (n = 19-60)	1.98 (1.26)	.92 (1.12)	.82 (1.06)	.76 (.93)	1.24 (1.26)	1.95 (1.36)	1.32 (1.32)
Mandibular basal (n = 19-60)	2.09 (1.32)	1.20 (1.30)	.92 (1.17)	.95 (1.12)	1.46 (1.34)	2.00 (1.35)	1.60 (1.30)

© 2020 Zimmerman S et al. *JAMA Network Open.*

eTable 4. Hierarchical Linear Mixed Model Estimates of Effects on Plaque and Gingival Oral Hygiene

Variables & Effects	Plaque Index for Long-Term Care			Gingival Index for Long-Term Care		
	**All Sextants** *n* = 1,126 β (SE); p	**Maxillary** *n* = 915 β (SE); p	**Mandibular** *n* = 1,105 β (SE); p	**All Sextants** *n* = 1,123 β (SE); p	**Maxillary** *n* = 912 β (SE); p	**Mandibular** *n* = 1,102 β (SE); p
**Intercept**	1.72 (.10); <.001	1.57 (.10); <.001	1.80 (.10); <.001	1.57 (.11); <.001	1.45 (.12); <.001	1.65 (.10); <.001
**Period (ref=Baseline)**						
4 months	−.78 (.06); <.001	−.85 (.07); <.001	−.76 (.07); <.001	−.75 (.07); <.001	−.75 (.08); <.001	−.76 (.07); <.001
12 months	−.96 (.06); <.001	−.92 (.07); <.001	−.97 (.07); <.001	−.92 (.07); <.001	−.87 (08); <.001	−.94 (.07); <.001
20 months	−.55 (.07); <.001	−.57 (.08); <.001	−.55 (.07); <.001	−.57 (.08); <.001	−.53 (.08); <.001	−.58 (.08); <.001
24 months	−.45 (.06); <.001	−.52 (.07); <.001	−.44 (.07); <.001	−.39 (.07); <.001	−.42 (.08); <.001	−.39 (.07); <.001
**Arm (ref=Mouth Care)**						
Control	−.10 (.15); .49	−.14 (.14); .31	−.08 (.15); .61	−.16 (.15); .30	−.17 (.17); .30	−.15 (.15); .33
**Interaction**						
24 months × Control	.35 (.09); <.001	.45 (.10); <.001	.35 (.10); <.001	.46 (.10); <.001	.50 (.11); <.001	.44 (.11); <.001
**Random Effects**						
Nursing home intercept	.06 (.03)	.05 (.02)	.06 (.03)	.05 (.03)	.07 (.03)	.05 (.03)
Resident intercept	.28 (.03)	.29 (.03)	.30 (.03)	.49 (.04)	.50 (.05)	.50 (.05)
Residual	.30 (.02)	.28 (.02)	.32 (.02)	.34 (.02)	.34 (.02)	.37 (.02)

© 2020 Zimmerman S et al. *JAMA Network Open.*

eTable 5. Two-Level Linear Mixed Model Estimates of Effects on Denture Plaque

**Variables & Effects**	**All Surfaces** *n* = 444 β (SE); p	**Maxillary Facial** *n* = 429 β (SE); p	**Maxillary Basal** *n* = 429 β (SE); p	**Mandibular Facial** *n* = 264 β (SE); p	**Mandibular Basal** *n* = 264 β (SE); p
**Intercept**	2.27 (.11); <.001	2.30 (.12); <.001	2.33 (.13); <.001	2.10 (.16); <.001	2.20 (.18); <.001
**Period (ref=Baseline)**					
4 months	−1.10 (.16); <.001	−1.27 (.17); <.001	−.88 (.19); <.001	−1.03 (.22); <.001	−.90 (.25); <.001
12 months	−1.28 (.17); <.001	−1.31 (.19); <.001	−1.37 (.21); <.001	−1.24 (.25); <.001	−1.25 (.28); <.001
20 months	−1.22 (.19); <.001	−1.44 (.20); <.001	−.96 (.22); <.001	−1.15 (.26); <.001	−1.10 (.30); <.001
24 months	−.64 (.15); <.001	−.79 (.17); <.001	−.47 (.18); .009	−.89 (.22); <.001	−.74 (.24); .002
**Arm (ref=Mouth Care)**					
Control	−.17 (.17); .31	−.25 (.18); .15	.06 (.19); .75	−.22 (.23); .34	−.26 (.24); .27
**Interaction**					
24 months × Control	.49 (.23); .032	.47 (.24); .053	.48 (.26); .070	.39 (.31); .21	.43 (.34); .21
**Random Effects**					
Resident intercept	.63 (.11)	.62 (.12)	.71 (.14)	.87 (.18)	.73 (.20)
Residual	.68 (.08)	.79 (.09)	.95 (.11)	.68 (.12)	.94 (.16)

© 2020 Zimmerman S et al. *JAMA Network Open.*

eFigure. Plaque, Gingival, and Denture Plaque Index Scores, Baseline Through 24 Months
(control data were collected at baseline and 24 months; line is extrapolated)

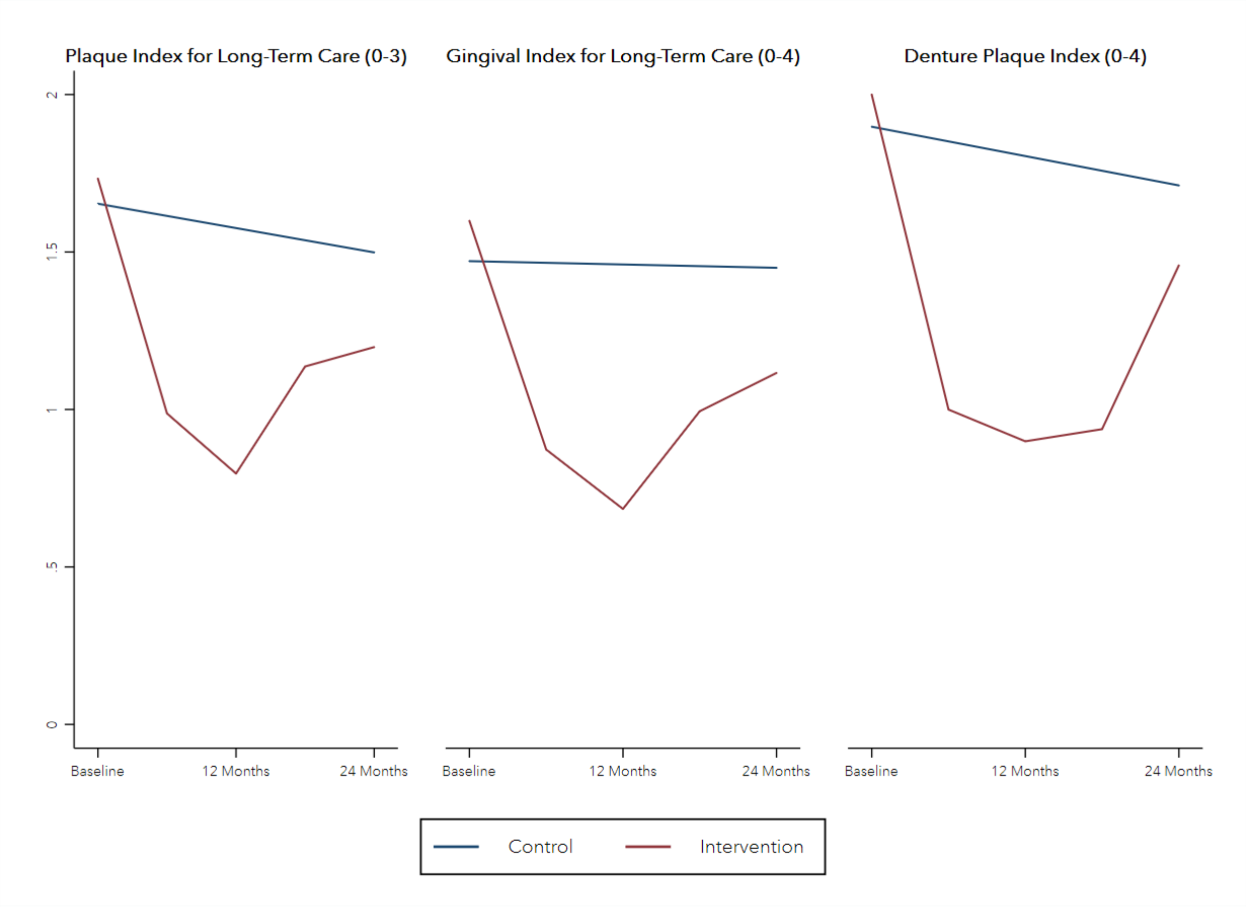

© 2020 Zimmerman S et al. *JAMA Network Open.*
