# Supplementary material for: Effectiveness of a Mouth Care Program Provided by Nursing Home Staff vs Standard Care on Reducing Pneumonia Incidence: A Cluster Randomized Trial
Source: JAMA Netw Open. 2020 Jun 19;3(6):e204321. doi: 10.1001/jamanetworkopen.2020.4321 (PMC7305523; doi:10.1001/jamanetworkopen.2020.4321)
Supplement: Supplement 3. — Data Sharing Statement [file jamanetwopen-3-e204321-s003.pdf]

Data Sharing Statement

Data
Data available: Yes
Data types: Deidentified participant data
How to access data: Send request to Sheryl_Zimmerman@unc.edu
When available: With publication

Supporting Documents
Document types: None

Additional Information
Who can access the data: Researchers who proposed use has been approved.
Types of analyses: For a specified purpose.
Mechanisms of data availability: With investigator support, after approval of proposal, with a signed data access agreement.
